# The Effects of Vitamin D on Muscle Strength Are Influenced by Testosterone Levels

**DOI:** 10.1002/jcsm.13733

**Published:** 2025-02-16

**Authors:** Aolin Yang, Qingqing Lv, Ziyu Han, Shimiao Dai, Yao Li, Mengru Hao, Ruirui Yu, Junying Zhu, Chenggang Yang, Zhan Shi, Ji‐Chang Zhou

**Affiliations:** ^1^ School of Public Health (Shenzhen) Shenzhen Campus of Sun Yat‐sen University Shenzhen Guangdong China; ^2^ Center for Synthetic Biochemistry, Shenzhen Institute of Synthetic Biology, Shenzhen Institutes of Advanced Technology Chinese Academy of Sciences Shenzhen Guangdong China; ^3^ Guangdong Province Engineering Laboratory for Nutrition Translation Sun Yat‐sen University Shenzhen Guangdong China; ^4^ Guangdong Provincial Key Laboratory of Food, Nutrition and Health Sun Yat‐sen University Guangzhou Guangdong China

**Keywords:** interaction, muscle strength, sarcopenia, sex difference, testosterone, vitamin D

## Abstract

**Background:**

Although the role of vitamin D receptor (VDR) in muscle mass and strength is well established, the effects of vitamin D (VD) on muscle remain controversial due to various factors. Herein, the influence of sex on the effects of VD on muscle function and the underlying reasons was explored.

**Methods:**

Male and female *Sod1* gene knockout (SKO) mice, serving as a model for skeletal muscle atrophy, were treated with the VD active analogue calcipotriol, and RNA sequencing was employed to investigate this potential signalling pathway. The National Health and Nutrition Examination Survey (NHANES) database was utilized to explore whether testosterone affects the correlation between VD and grip strength in human participants. Experiments involving C2C12 cells and castrated male mice subjected to immobilization were conducted to demonstrate the enhancing effects of testosterone on VD function.

**Results:**

In male SKO mice, *Vdr* expression in the gastrocnemius muscle was positively correlated with grip strength (*R* ^2^ = 0.4689, *p* < 0.001), whereas no such correlation was identified in female mice. At 28 weeks of age, both male and female SKO mice exhibited significantly reduced grip strength compared to *Sod1* wild‐type (SWT) mice, and calcipotriol restored grip strength in male SKO mice (SWT‐veh: 0.0716 ± 0.0006, SWT‐cal: 0.0686 ± 0.0010, SKO‐veh: 0.0601 ± 0.0010, SKO‐cal: 0.0703 ± 0.0007; *p* < 0.05). Calcipotriol increased muscle protein synthesis and mitochondrial biogenesis while decreasing inflammation and atrogenes in gastrocnemius muscle of male SKO mice. However, the effect of calcipotriol on muscle was not significant in female SKO mice. Compared to wild‐type mice, both male and female SKO mice exhibited reduced levels of 1,25(OH)_2_D_3_ due to ROS‐induced hepatic CYP3A4 overexpression, thereby excluding the influence of baseline VD levels. The serum 25(OH)D_3_ and testosterone interactively affect grip strength in adults (*p* < 0.05). Using C2C12 cells differentiated into myotubes, testosterone significantly enhanced the inducing effects of VD on VDR, androgen receptor (AR), P‐AKT, PGC1α, Beclin1 and LC3B. Calcipotriol improved grip strength in sham‐operated mice but had a negligible effect on grip strength in castrated mice. However, a significant improvement in grip strength was observed in castrated mice following testosterone restoration (*p* < 0.05).

**Conclusions:**

This study demonstrates the existence of sex heterogeneity in the effects of VD on muscle and that testosterone enhances the strength and molecular responses to VD. These findings underscore the importance of considering testosterone levels when utilizing VD to enhance muscle strength.

## 1 Introduction

Skeletal muscle, comprising approximately 40%–50% of the body mass, plays a crucial role in movement and metabolism [1]. Skeletal muscle mass and performance decline with aging, disuse, denervation or cachexia, leading to reduced mobility and an elevated risk of falls [2, S1]. Vitamin D (VD) is a fat‐soluble steroid hormone. Vitamin D_3_ (VD_3_) undergoes hydroxylation by the principal VD 25‐hydroxylase CYP2R1 in the liver to produce 25‐hydroxyvitamin D_3_ [25(OH)D_3_]. This is followed by secondary hydroxylation by the 1α‐hydroxylase CYP27B1 in the proximal renal tubules, forming 1,25‐dihydroxyvitamin D_3_ [1,25(OH)_2_D_3_]. Finally, it is inactivated through 24‐hydroxylation by CYP24A1 in the kidneys [3]. Additionally, in non‐classical metabolic pathways, CYP3A4 in the liver can hydroxylate and inactivate 1,25(OH)_2_D_3_ at the 23‐position [4]. VD deficiency is a prevalent global health issue, particularly affecting elderly populations [5]. Observational studies have shown a positive correlation between VD and muscle strength [6]. VD exerts its transcriptional regulatory and rapid signalling functions through the ligand‐receptor‐dependent form via the vitamin D receptor (VDR) [7]. The beneficial effect of VDR on muscle strength has been fully elucidated by both myocyte‐specific VDR knockout [8] and muscle VDR overexpression mouse models [9].

However, monotherapy with VD has no significant beneficial effect on muscle performance in adults [10]. Various factors can influence the effect of VD on muscle function. Previous studies have found that the effects of VD on muscle are influenced by baseline VD levels and physical activity levels [11, 12]. Recent cross‐sectional studies have suggested a sex difference in the association between VD and sarcopenia, with a stronger correlation observed in males [13, S2, S3]. However, the causal relationship between sex and the association of VD with sarcopenia, as well as the mechanisms underlying this difference, remains unclear.

The accumulation of reactive oxygen species (ROS) contributes to skeletal muscle atrophy via the induction of atrophy programming [14]. Cu/Zn‐superoxide dismutase (*Sod1*) gene knockout (SKO) mice are used as a model for studying sarcopenia and frailty [15, 16], as the accumulation of endogenous ROS promotes ageing. In this study, we found that treatment with the VD active analogue, calcipotriol, a VDR ligand, can rejuvenate grip strength in male SKO mice, but it did not affect female mice. To investigate the reasons behind the sex heterogeneity of VD's effects on muscle strength, we utilized the National Health and Nutrition Examination Survey (NHANES) database and conducted studies on castrated male mice. Our findings indicated that testosterone enhances grip strength and molecular response to the VD.

## 2 Materials and Methods

### 2.1 Animals

All animal experiments were conducted following the guidelines and were approved by the ethical approval of the Experimental Animal Ethics Committee, SUN YAT‐SEN UNIVERSITY (no. SYSU‐IACUC‐MED‐2020‐B0001). All mice were maintained group‐housed under specific pathogen‐free (SPF) conditions with a 12‐h/12‐h light–dark cycle at 23°C with free access to food and water at the Laboratory Animal Center of Sun Yat‐sen University, China.

Cu/Zn‐superoxide dismutase gene (*Sod1*) knockout mice were purchased from The Jackson Laboratory (B6;129S‐Sod1^tm1Leb^/J, JAX:002972, Bar Harbor, ME, USA). By crossing heterozygous mice, the homozygous SKO mice and their littermate homozygous *Sod1* wild‐type (SWT) mice were obtained and used for the experiment. The male C57BL/6J mice were purchased from BesTest Bio‐Tech Co. Ltd. (C57X5W, Zhuhai, Guangdong, China) for the immobilization model experiment.

### 2.2 Cells

The cell lines used in this study were C2C12 (sex unknown) (CRL‐1772), HepG2 (male) (HB‐8065) and 293T (sex unknown) (CRL‐3216). These cells were purchased from ATCC (Manassas, VA, USA). Primary hepatocytes (male) isolated from SKO and SWT mice at 8 weeks of age in house (described below). Specific conditions for cell culture can be found in the Supplemental Methods.

### 2.3 Human Participants

Cross‐sectional data were drawn from the NHANES (https://www.cdc.gov/nchs/nhanes/) from 2011 to 2014. Individuals who had combined grip strength and serum 25(OH)D_3_ were included. The exclusion criteria were as follows: taking supplements of VD (D_2_ or/and D_3_) and aged under 18 years. In addition, missing data were excluded. Ultimately, a total of 5707 eligible participants were included in the study. A full description of survey protocols and all data are available at the US Centers for Disease Control (CDC) NHANES website.

### 2.4 In Vivo Treatments

For the calcipotriol treatment study, 6‐week‐old SKO and SWT mice including both sexes were randomly divided into four groups. After 1 week of adaption, each group was injected intraperitoneally (*i.p*.) with calcipotriol (HY‐10001, MCE, Monmouth Junction, NJ, USA) (60 μg/kg body weight) [15], or vehicle three times a week, with the intervention lasting until 28 weeks [17]. *n* = 7 each for male mice; *n* = 6–8 for female mice.

For the immobilization model study, male C57BL/6J wild‐type mice underwent castration or sham surgery at 5 weeks old, followed by random grouping at 7 weeks old. After an acclimation period of 1 week, the mice were subcutaneous injections (*s.c*.) of testosterone enanthate (TSTE, T) (C6457, ApexBio, Houston, TX, USA) (0.9 mg/mouse, once a week) [S4] and were administered *i.p*. with calcipotriol (60 μg/kg body weight, three times a week) or vehicle alone or in combination. Subsequently, the mice underwent immobilization (IM) or non‐immobilization (NIM) treatment for 1 week (*n* = 6–10).

After treatment, mice were fasted overnight (12 h), anaesthetized with pentobarbital sodium and euthanized by cervical dislocation after cardiac puncture for blood collection. The gastrocnemius muscle, liver and kidneys were dissected and quickly frozen in liquid nitrogen before being stored at −80°C. The left hind limb gastrocnemius muscle of each mouse was fixed with 4% paraformaldehyde buffer for histological analysis.

### 2.5 In Vitro Treatments

HepG2 cells were seeded for 24 h and treated with 4NQO (N8141, Sigma, St Louis, MO, USA) (1 μM) for 24 h. After pre‐treatment with PF‐4981517 (PF, GC12280, GlpBio, Montclair, CA, USA) (10 μM) or vehicle for 2 h, the old medium was replaced with fresh medium containing varying concentrations of 1,25(OH)_2_D_3_ (HY‐10002, MCE, Monmouth Junction, NJ, USA) (0.1, 1, 10 nM) or vehicle for 2 h. The supernatant from the HepG2 culture was then used to incubate C2C12 cells for 48 h to measure VDR expression.

The C2C12 cells were seeded in a six‐well plate and induced to differentiate when they reached over 70% confluence with 2% horse serum. During the differentiation process, the cells were treated every 2 days with either 1,25(OH)_2_D_3_ (10 nM) [S5] or calcipotriol (10 nM), testosterone (GC45935, GlpBio, Montclair, CA, USA) (TST, 10 nM, approximately 300 ng/dL) or vehicle, continuing for a total of 8 days.

### 2.6 Grip Strength Measurements

Grip strength measurements were done using the HANDPI HP‐5N grip strength meter. Each group was measured at the same time and by the same investigator who was blinded for treatment. Each mouse was measured with four paws on the grid five times with at least 5 s of recovery between measurements. Removing the maximum and minimum values, the average of the three measurements was used for further analyses.

### 2.7 Surgical Castration

Castration of male mice was performed using the bilateral orchiectomy method through the scrotum [S6]. Additional details are provided in the Supplemental Methods.

### 2.8 Serum Measurements

Serum 1,25(OH)_2_D_3_, calcium and phosphate levels were measured using commercial assay kits. Serum 25(OH)D_3_ was determined by liquid chromatography–tandem mass spectrometry. Additional details are provided in the Supplemental Methods.

### 2.9 Quantitative PCR

The experimental methods were previously described [17] and are detailed in the Supplemental Methods. Primer sequences can be found in Table S5.

### 2.10 Transcriptomic Analysis

RNA sequencing (RNA‐seq) was performed on the gastrocnemius muscles of four groups of male mice treated with calcipotriol or vehicle (*n* = 3 each). Further details can be found in the Supplemental Methods.

### 2.11 Histology and Image Analysis

To assess muscle fibre cross‐sectional area (CSA) and quantity, haematoxylin and eosin (H&E) staining and immunofluorescent staining for myosin heavy chain 2b (MHC2b) were employed. Detailed staining and analysis methods are provided in the Supplemental Methods.

### 2.12 Silver Stain

Skeletal muscle myosin heavy chain (MHC) composition was determined by distinguishing all four MHC isoforms, MHC2a, MHC2x, MHC2b and MHC1 as described previously [S7]. Additional details are available in the Supplemental Methods.

### 2.13 Western Blotting

The experimental methods were previously described [12] and are detailed in the Supplemental Methods. For antibodies, see Table S6.

### 2.14 Dual‐Luciferase Reporter Assays

The interaction between ATF3 and the *CYP3A4* gene promoter region was assessed using a dual‐luciferase reporter gene system, as detailed in the Supplementary Methods.

### 2.15 Quantification and Statistical Analysis

For mouse and cell experiments, the results are presented as the mean and standard error of the mean (mean ± SEM). Comparisons of two groups were done using Student's unpaired two‐tailed *t*‐test and for more than two groups, one or two‐way ANOVA (two‐tailed) followed by Tukey's post hoc test for multiple comparisons, respectively. Linear regression models were used to verify the linear correlation between variables. Linear regression analysis was done using an *F* test. A *p* < 0.05 was considered statistically significant. All statistical analyses were performed using Prism v9.4.1 (GraphPad). For the observational study, database analyses were performed in R (Version 4.3.1). To detect the interaction of serum 25(OH)D_3_ and testosterone on handgrip strength, the interaction terms from all linear regression equations were computed. Additional details are available in the Supplemental Methods.

## 3 Results

### 3.1 The Level of *Vdr* in Gastrocnemius Muscle Is Positively Associated With Grip Strength in Male Mice

**FIGURE 1:**
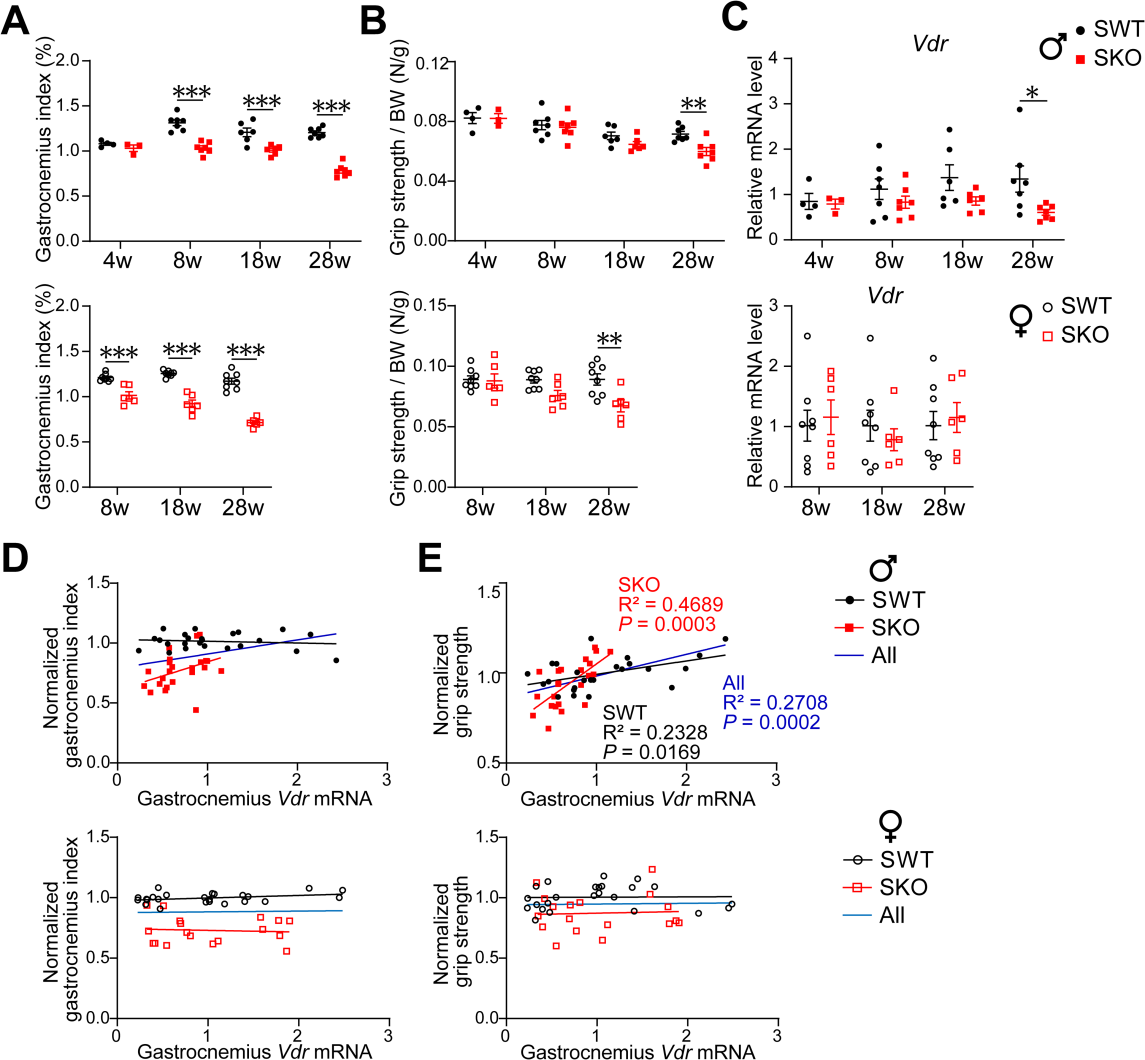
The level of *Vdr* in the gastrocnemius muscle is positively correlated with grip strength in male mice. (A–E) Male SWT and SKO mice aged 4 (*n* = 3–4), 8 (*n* = 7), 18 (*n* = 6) and 28 (*n* = 7) weeks. Female SWT and SKO mice aged 8, 18 and 28 weeks (*n* = 6–8). The 28‐week‐old mice were treated with a vehicle as indicated by Figure 2A showing the experimental scheme. (A,B) Gastrocnemius mass (A) and grip strength (B) of male and female mice. (C) mRNA levels of *Vdr* in gastrocnemius of male and female mice. (D,E) Correlations with regression lines to examine the relationship between gastrocnemius *Vdr* mRNA level and muscle mass (D) or grip strength (E) of male and female mice. Gastrocnemius index = gastrocnemius mass/body weight × 100%. Data are represented as mean ± SEM. *p < 0.05, **p < 0.01 and ***p < 0.001 by two‐way ANOVA (two‐tailed) with Tukey's post hoc for multiple comparisons or simple linear regression.

Both male and female SKO mice exhibited a decrease in gastrocnemius muscle mass as early as 8 weeks, with differences in grip strength relative to body weight evident at 28 weeks (Figure 1A,B). The mRNA levels of *Vdr* decreased in the gastrocnemius at 28 weeks of male SKO mice, but not in females (Figure 1C). Additionally, gastrocnemius *Vdr* mRNA levels are positively associated with the grip strength of male mice, particularly in SKO mice (*R* ^2^ = 0.4689), although no correlation was found with muscle mass in either SKO or SWT mice (Figure 1D,E). However, the associations of gastrocnemius *Vdr* mRNA levels with both muscle mass and grip strength were not observed in female mice.

### 3.2 Calcipotriol Rejuvenates Grip Strength in SKO Male Mice

**FIGURE 2:**
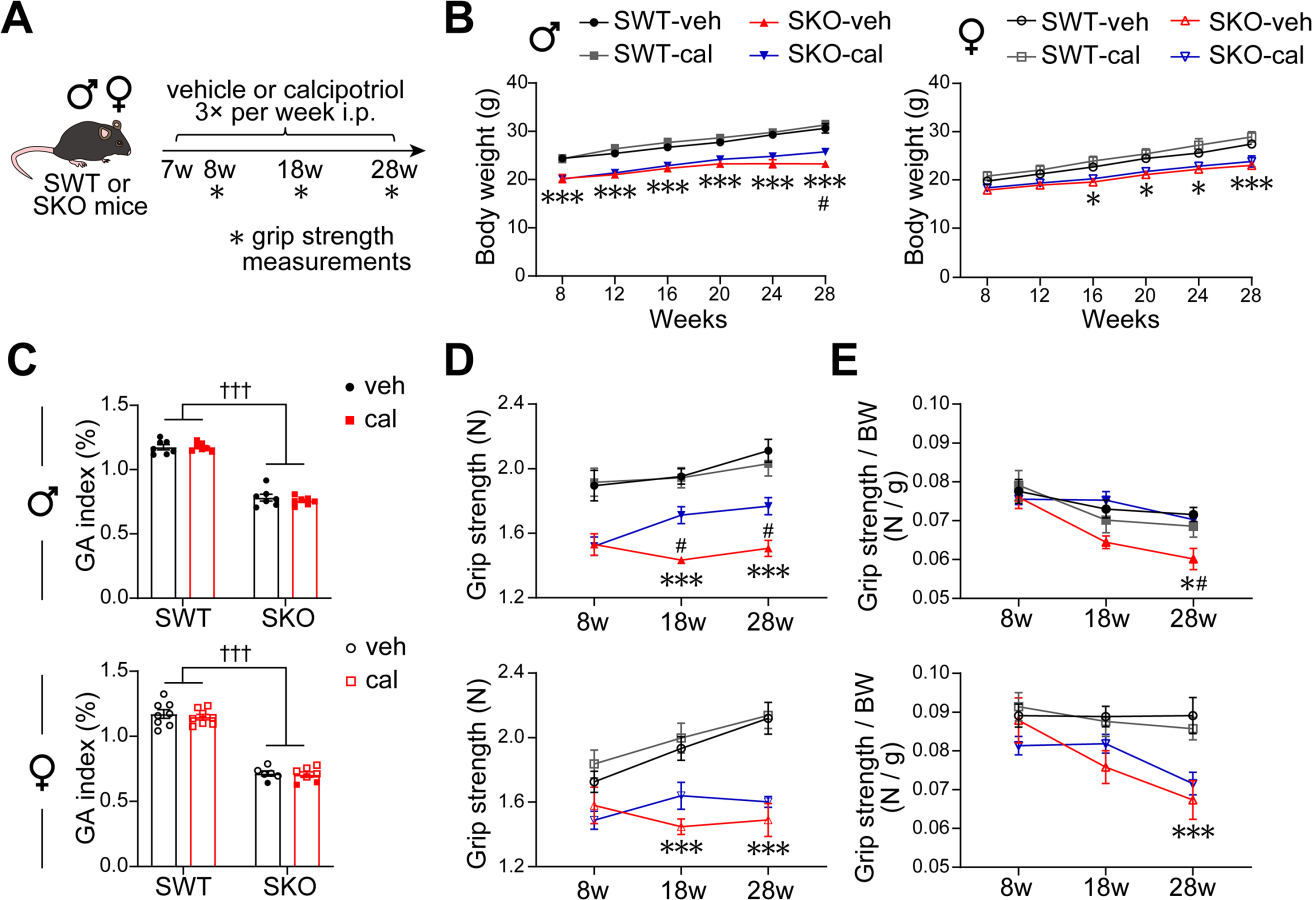
Calcipotriol rejuvenates grip strength in male SKO mice. (A) Experimental scheme. (B) Body weight of different weeks of age. (C–E) Weight of dissected gastrocnemius (C), grip strength (D) and grip strength normalized to body weight (E) measured at 8, 18 and 28 weeks old in mice. Data are represented as mean ± SEM. #,*p < 0.05 and ***,††† *p* < 0.001 by two‐way ANOVA (two‐tailed) with Tukey's post hoc test to determine individual group differences. *SWT‐veh vs. SKO‐veh; #SKO‐veh vs. SKO‐cal; †main effect of SWT vs. SKO. cal, calcipotriol; GA, gastrocnemius; veh, vehicle.

Calcipotriol was administered to both male and female SWT and SKO mice to investigate potential sex differences in the effects of VD on muscle function (Figure 2A). Calcipotriol significantly increased body weight in male SKO mice (Figure 2B), and rejuvenated grip strength, but did not affect muscle mass (Figure 2C–E). However, no effect on body weight, grip strength or muscle mass was observed in female mice.

### 3.3 Calcipotriol Increases MHC2b‐Type Myofibres in Male SKO Mice

**FIGURE 3:**
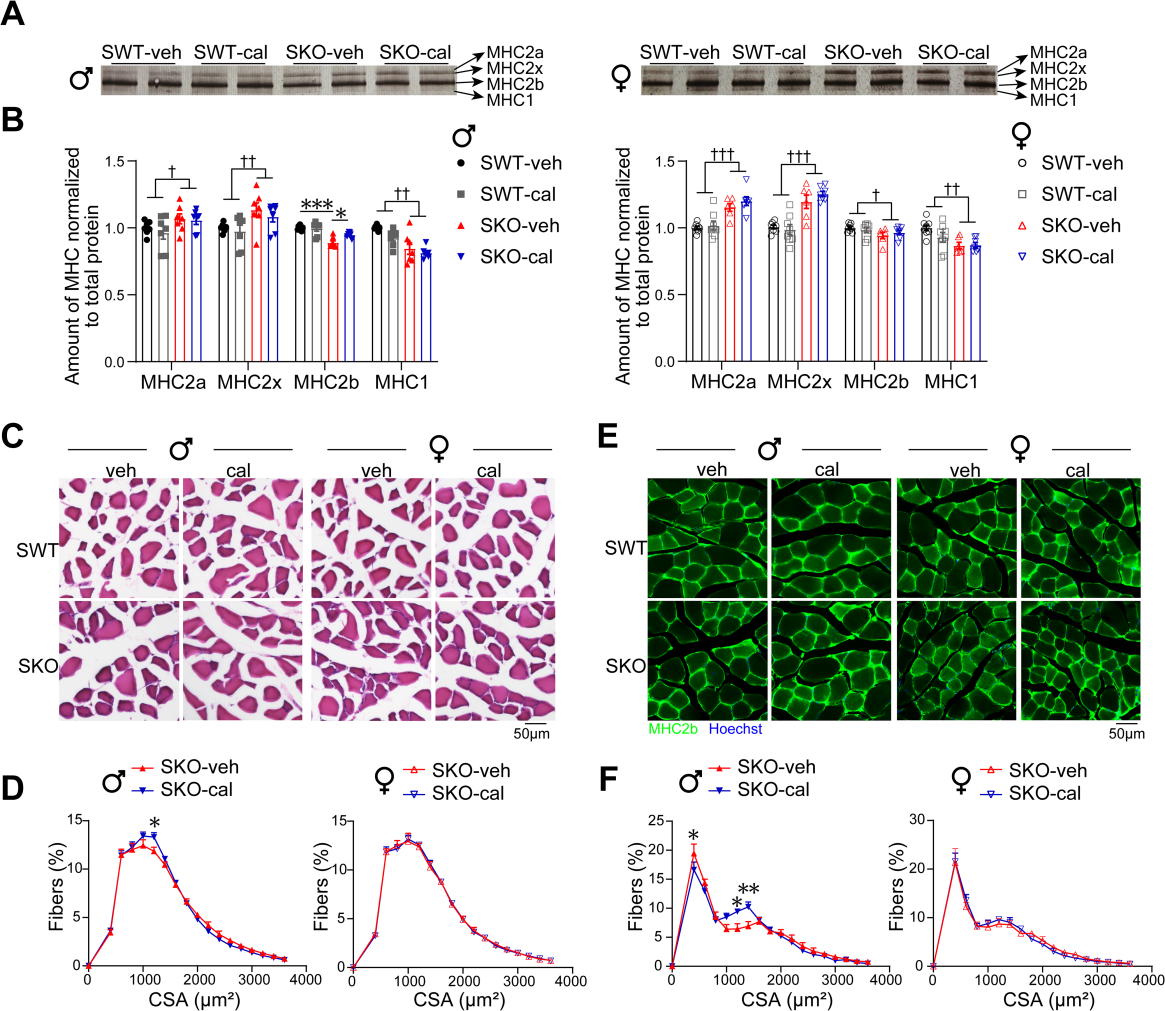
Calcipotriol increases MHC2b‐type myofibres in male SKO mice. (A,B) Representative gastrocnemius muscle MHC isoform levels by silver stain (A) and analysis grayscale in each group of male and female mice (B). (C,D) Representative gastrocnemius cross section by H&E stains (C). Cross‐sectional area analysis in SKO‐veh and SKO‐cal of males and females (D). Scale bars, 50 μm. (E,F) Representative gastrocnemius cross section by MHC2b stains (E). Cross‐sectional area analysis in SKO‐veh and SKO‐cal of male and female mice (F). Scale bars, 50 μm. Data are represented as mean ± SEM. #,*,†*p* < 0.05, **,††*p* < 0.01 and ***,†††*p* < 0.001 by two‐way ANOVA (two‐tailed) with Tukey's post hoc for multiple comparisons. † Main effect of SWT vs. SKO. cal, calcipotriol; CSA, cross‐sectional areas; GA, gastrocnemius; veh, vehicle.

Protein silver staining of MHC isoforms revealed an increase in the levels of MHC2a and MHC2x and a decrease in the levels of MHC2b and MHC1 in SKO mice. Additionally, calcipotriol increased the level of MHC2b in male SKO mice (Figure 3A,B). Similarly, calcipotriol increased the level of *Myh4* mRNA (encoding MHC2b), but it did not affect *Myh1* (encoding MHC2x), *Myh2* (encoding MHC2a) and *Myh7* (encoding MHC1) (Figure S1A,B). Statistical analysis of gastrocnemius H&E staining revealed a reduction in the number of myofibres in SKO mice. Treatment with calcipotriol increased the proportion of myofibres with a CSA of 1000–1200 μm^2^ in SKO male mice, whereas it did not affect female mice (Figures 3C,D and S1C,D). Staining for MHC2b revealed a significant reduction in the CSA of MHC2b fibres in SKO mice. Calcipotriol decreased the proportion of fibres with a CSA of 200–400 μm^2^ and increased the proportion of fibres with a CSA of 1000–1400 μm^2^ in male SKO mice, whereas it did not affect female mice (Figures 3E,F and S1E,F).

### 3.4 Calcipotriol Increases Muscle Protein Synthesis in Male SKO Mice

**FIGURE 4:**
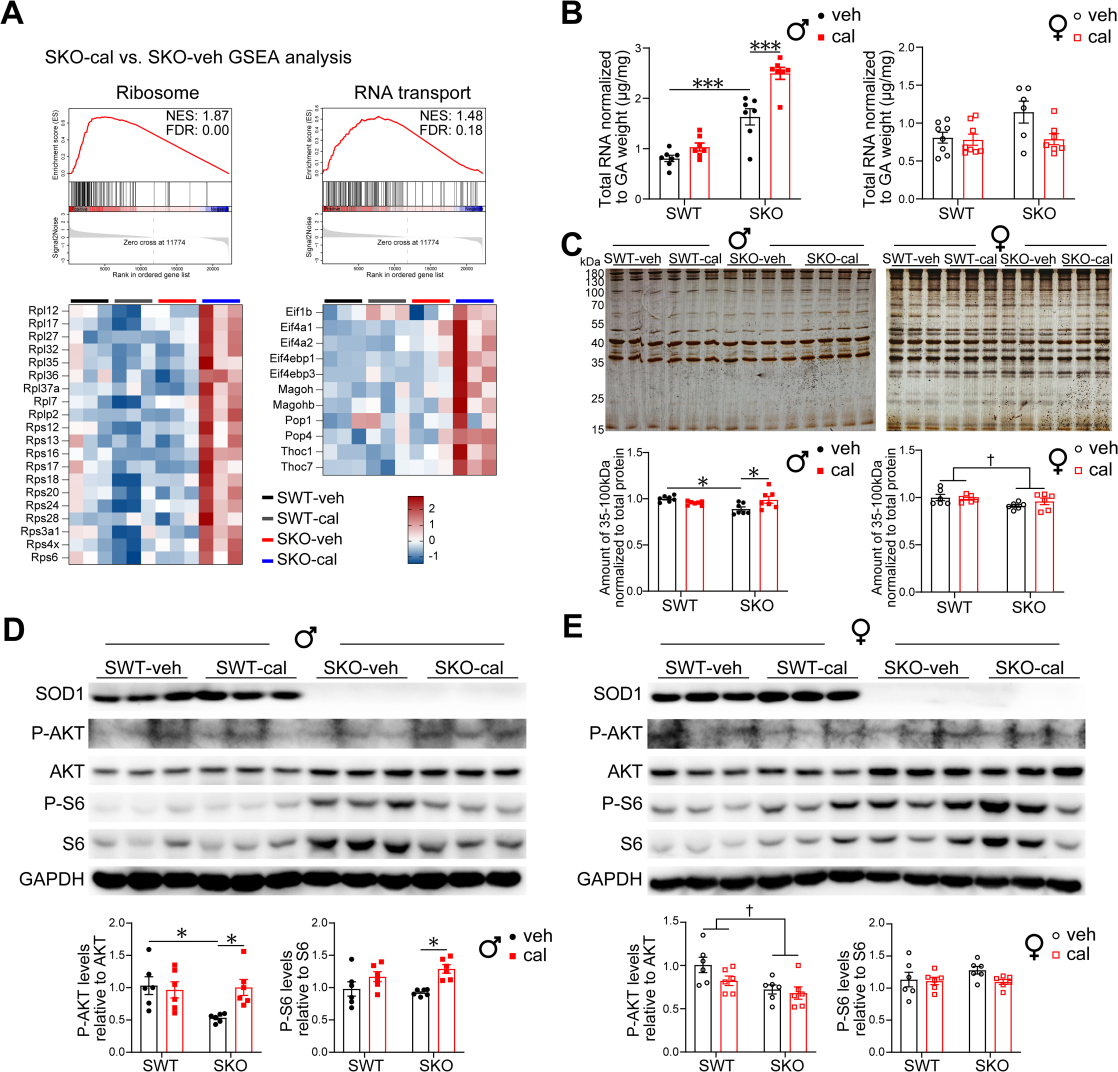
Calcipotriol increases muscle protein synthesis in male SKO mice. (A) GSEA showing ribosome and RNA transport pathways up‐regulated in SKO‐cal compared to SKO‐veh male mice. Heat maps show the expression of the core genes that contribute to pathway enrichment (*n* = 3 each). (B) Expression level of gastrocnemius muscle total RNA yield (*n* = 6–8). (C) Representative gastrocnemius muscle protein level by silver stain, and analysis 35–100 kDa grayscale in each group of male and female mice (*n* = 6–8). (D,E) Representative western blotting results showing the analysis of SOD1, P‐AKT, total AKT, P‐S6 and total S6 in gastrocnemius of male (D) and female (E) mice (*n* = 6 biological replicates per experimental group). Data are represented as mean ± SEM. *,† *p* < 0.05 and ***p < 0.001 by two‐way ANOVA (two‐tailed) with Tukey's post hoc for multiple comparisons. †Main effect of SWT vs. SKO. cal, calcipotriol; GSEA, Gene Set Enrichment Analysis; NES, normalized enrichment score; veh, vehicle.

Using RNA‐seq approach, we identified 30 genes that exhibited significant differences in response to calcipotriol treatment in SWT mice, whereas 8 genes showed significant differences in SKO mice (Figure S2A). Due to the limited number of significantly different genes, we conducted a further analysis with Gene Set Enrichment Analysis (GSEA). The findings demonstrated an increase in the proteasome pathway in the muscle of SKO mice, whereas the ribosome and RNA transport pathways were up‐regulated following calcipotriol intervention (Figures 4A and S2B). Following calcipotriol intervention, there was an increase in total RNA abundance in SKO mice (Figure 4B). Silver staining of total protein revealed a decrease in protein levels in the gastrocnemius muscles of SKO mice, whereas calcipotriol treatment led to an increase in protein levels with a molecular weight ranging from 35 to 100 kDa in male SKO mice (Figure 4C). In male SKO mice, calcipotriol increased the levels of P‐AKT and decreased total S6. However, calcipotriol had no significant effect on P‐AKT and S6 in female SKO mice (Figure 4D,E).

### 3.5 Calcipotriol Inhibits Inflammation and Atrogenes in Male SKO Mice

**FIGURE 5:**
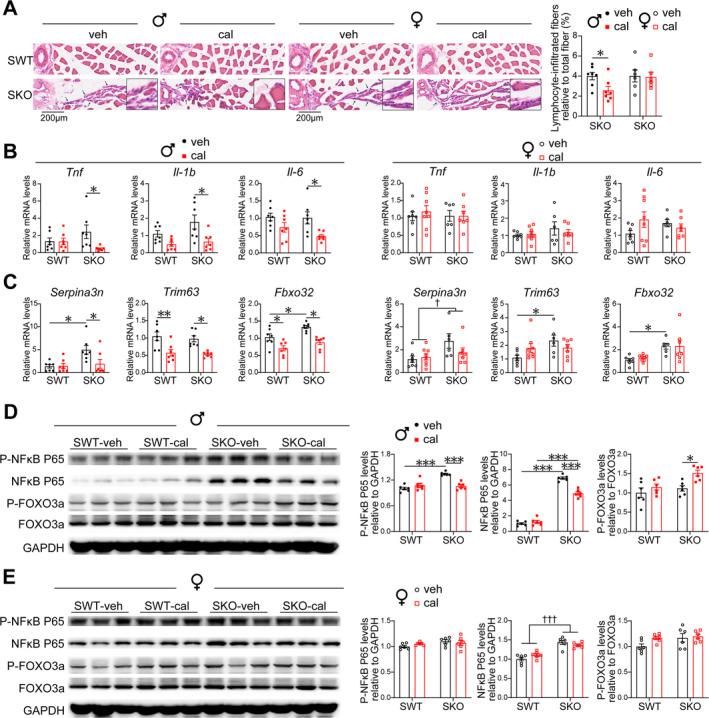
Calcipotriol inhibits inflammation and atrogenes in male SKO mice. (A) Representative gastrocnemius cross section by H&E stains. The summary plot shows lymphocyte‐infiltrated fibres count in SKO‐veh and SKO‐cal of male and female mice (*n* = 6–7). Scale bars, 200 μm. Black arrow indicates lymphocyte infiltration. (B,C) mRNA levels of gastrocnemius inflammatory genes (B) and atrogenes (C) in male and female mice (*n* = 6–8). (D,E) Representative western blotting results showing the analyses of P‐FOXO3a, total FOXO3a, P‐NFκB P65 and total NFκB P65 in gastrocnemius of male (D) and female (E) mice (*n* = 6 biological replicates per experimental group). Data are represented as mean ± SEM. *,† *p* < 0.05, **p < 0.01 and ***,††† *p* < 0.001 by Student's unpaired two‐tailed *t*‐test, or by two‐way ANOVA (two‐tailed) with Tukey's post hoc for multiple comparisons. † Main effect of SWT vs. SKO. cal, calcipotriol; veh, vehicle.

H&E staining revealed the infiltration of lymphocytes in the interstitium surrounding blood vessels and between muscle fibres in SKO mice. Calcipotriol reduced the infiltration of lymphocytes within muscle fibres (Figure 5A). Based on the RNA‐seq results, we validated the expression of several inflammatory factor genes. Calcipotriol decreased the expression levels of *Il‐1b*, *Il‐6* and *Tnf* in male SKO mice (Figure 5B). P‐NFκB p65 was increased in male SKO mice, but this increase was reversed by calcipotriol treatment (Figure 5D,E). We examined the expression of muscle differentiation marker genes and found that the levels of *Myod1*, *Myog*, *Myf5* and *Myf6* were up‐regulated in SKO mice and calcipotriol increased the levels of *Myod1*, *Myog* and *Myf6* in male SKO mice (Figure S2C,D). The effects of calcipotriol are not shown in female SKO mice.

Serpina3n is a secreted serine protease inhibitor, and increased levels of Serpina3n are associated with cachexia and sarcopenia, serving as an indicator of inflammation levels [18]. In male SKO mice, calcipotriol decreased the mRNA levels of *Serpina3n* genes (Figure 5C). Trim63 and Fbox32 are muscle‐specific E3 ubiquitin‐protein ligases that serve as indicative markers of muscle atrophy, and the expression of these genes is primarily regulated by FOXO3a [19]. In male SKO mice, calcipotriol decreased the expression levels of *Trim63* and *Fbox32* (Figure 5C) while enhancing the phosphorylation level of FOXO3a. However, calcipotriol did not cause those results in female SKO mice (Figure 5D,E).

### 3.6 Calcipotriol Boosts Mitochondrial Biogenesis and Oxidative Phosphorylation in Male SKO Mice

**FIGURE 6:**
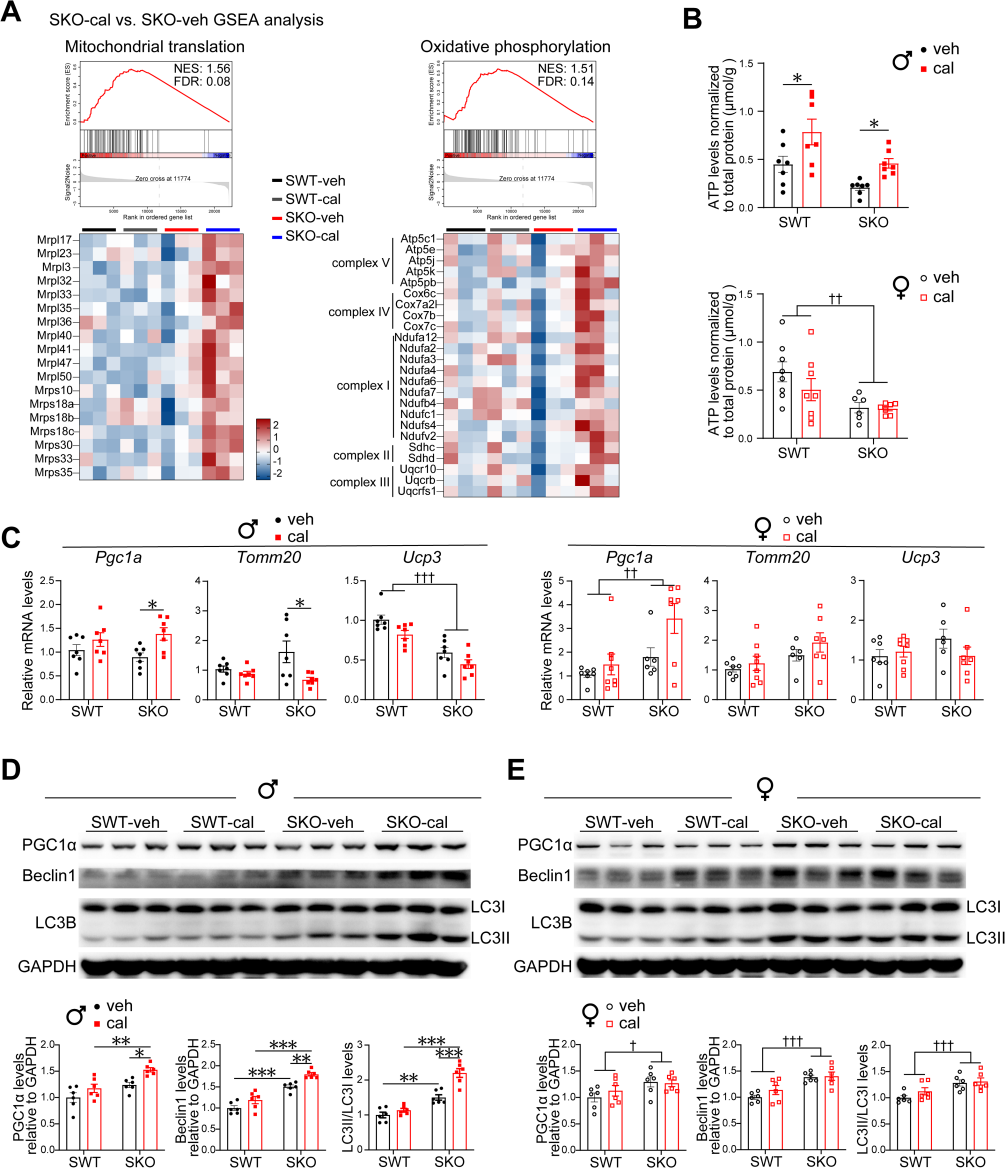
Calcipotriol boosts mitochondrial biogenesis and oxidative phosphorylation in male SKO mice. (A) GSEA showing mitochondrial translation and oxidative phosphorylation pathways up‐regulated in SKO‐cal compared to SKO‐veh male mice. Heat maps showing expressions of the core genes that contribute to pathway enrichment (*n* = 3). (B) ATP levels in gastrocnemius muscle (*n* = 6–8). (C) mRNA levels of gastrocnemius *Pgc1a*, *Tomm20* and *Ucp3* in male and female mice (*n* = 6–8). (D,E) Representative western blotting results showing the analysis of PGC1α, Beclin1 and LC3B in gastrocnemius of male (D) and female (E) mice (*n* = 6 biological replicates per experimental group). Data are represented as mean ± SEM. *,†*p* < 0.05, **,††*p* < 0.01 and ***,†††*p* < 0.001 by two‐way ANOVA (two‐tailed) with Tukey's post hoc for multiple comparisons. † Main effect of SWT vs. SKO. cal, calcipotriol; veh, vehicle.

RNA‐seq analysis indicated that calcipotriol treatment up‐regulates the mitochondrial translation and oxidative phosphorylation pathway (Figure 6A). Calcipotriol increased the level of ATP in the gastrocnemius muscle of male mice (Figure 6B). PGC1α serves as a crucial regulator of mitochondrial biogenesis [S8]. Calcipotriol elevated the expression levels of PGC1α mRNA and protein in male SKO mice (Figure 6C–E). Calcipotriol reduced the levels of *Tomm20* but did not have a significant effect on *Ucp3* (Figure 6C). To maintain a functional mitochondrial population, damaged or stressed mitochondria undergo degradation and clearance through autophagy. Calcipotriol enhanced the protein expression of autophagy markers, LC3II/LC3I and Beclin1 (Figure 6D,E). However, calcipotriol treatment still showed no effect on mitochondrial function in female mice.

### 3.7 Serum 1,25(OH)_2_D_3_ Level Decreases in Both Male and Female SKO Mice

**FIGURE 7:**
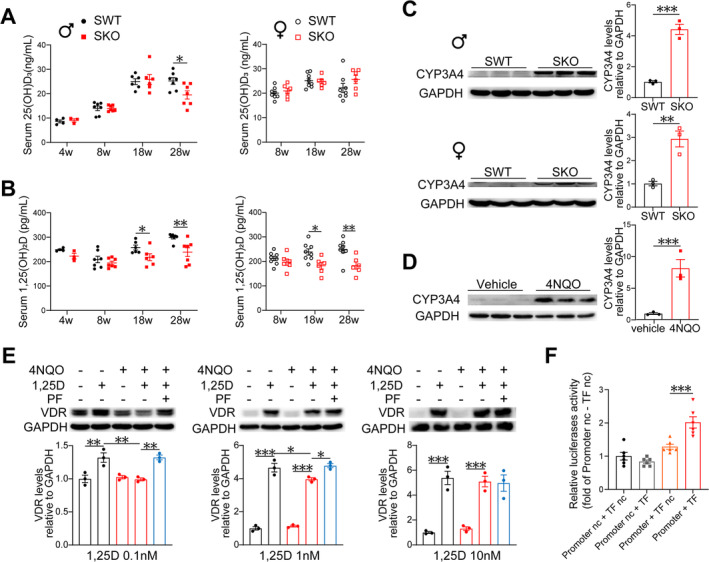
ROS‐induced overexpression of CYP3A4 accelerates the depletion of 1,25(OH)_2_D_3_. (A,B) Serum 25(OH)D_3_ and 1,25(OH)_2_D_3_ levels in male and female mice (*n* for each group same as Figure 1A). (C) Expression levels of CYP3A4 in male and female primary hepatocytes isolated from SWT and SKO mice at 8 weeks of age (*n* = 3). (D) Expression levels of CYP3A4 protein at 48 h in HepG2 cells treated with 4NQO (1 μM) or vehicle (*n* = 3). (E) Expression level of VDR by western blot in C2C12 cells (*n* = 3 biological replicates per experimental group). (F) Analysis of the dual‐luciferase reporter gene. After transfection of 293T cells with the luciferase promoter plasmid, ATF3 overexpression plasmid (TF), or promoter negative control plasmid (nc), TF nc plasmid, luciferase activity assay was conducted at 48 h (*n* = 6). Data are represented as mean ± SEM. *p < 0.05, **p < 0.01 and ***p < 0.001 by Student's unpaired two‐tailed *t*‐test, or by one‐way ANOVA (two‐tailed) with Tukey's post hoc for multiple comparisons. 1,25D, 1,25(OH)_2_D_3_; PF, PF‐4981517 (CYP3A4 specific inhibitor); veh, vehicle.

The decreased level of *Vdr* mRNA in gastrocnemius (Figure 1C) suggested a VD deficiency in SKO mice. The levels of serum 1,25(OH)_2_D_3_ decreased in both male and female SKO mice at 18 and 28 weeks, whereas serum 25(OH)D_3_ levels decreased at 28 weeks in male SKO mice but remained unchanged in females (Figure 7A,B). The level of *Cyp24a1* decreased in the kidneys at 8, 18 and 28 weeks in male SKO mice, *Vdr* decreased at 18 and 28 weeks, and *Cyp27b1* levels increased at 4 and 8 weeks (Figure S3A). Additionally, in 28‐week‐old female SKO mice, *Vdr* and *Cyp24a1* levels were decreased, whereas *Cyp27b1* levels were increased, mirroring the findings observed in male mice. In both male and female mice, calcipotriol decreased liver *Cyp2r1* levels and serum 25(OH)D_3_ levels while increasing *Cyp24a1* levels and decreasing *Cyp27b1* levels in the kidneys (Figure S4A,E,F). However, it did not affect serum 1,25(OH)_2_D_3_ levels and did not significantly affect calcium and phosphorus homeostasis (Figure S4B–D). This may be attributed to calcipotriol having an affinity for the VDR comparable to that of 1,25(OH)_2_D_3_, although its shorter serum half‐life results in far less disturbance to calcium and phosphorus metabolism compared to 1,25(OH)_2_D_3_ [20]. In summary, the reduced serum 1,25(OH)_2_D_3_ levels observed in both male and female SKO mice may be due to metabolic processes involving other pathways.

### 3.8 ROS Induces Overexpression of CYP3A4 in Hepatocytes

Previous studies have reported that CYP3A4 is responsible for metabolizing 1,25(OH)_2_D_3_ into inactive metabolites [21]. In our RNA‐seq data from the livers of SKO mice, we observed a significant increase in the levels of *Cyp3a41* (human *CYP3A4* homologue) by approximately 100‐ to 2000‐fold as measured by real‐time quantitative PCR (qPCR) (Figure S3B). The protein levels in primary hepatocytes of male and female mice increased approximately 3‐ to 4‐fold as determined by western blot using a human CYP3A4 antibody (Figure 7C). Treatment of WT mice with 4‐nitroquinoline‐N‐oxide (4NQO, a superoxide inducer) increased the *Cyp3a41* abundance in the liver while decreasing *Cyp24a1* and *Vdr* abundances and increasing the *Cyp27b1* abundance in the kidneys (Figure S3C,D). 4NQO increased CYP3A4 mRNA levels by 15‐fold and protein expression by 8‐fold in HepG2 cells (Figure S3E and Figure 7D). Tempol is an SOD‐mimetic drug that efficiently neutralizes ROS. Tempol inhibited the level of 4NQO‐induced *CYP3A4* (Figure S3F).

### 3.9 ROS‐Induced CYP3A4 Inhibits the Induction of VDR by 1,25(OH)_2_D_3_

To investigate the impact of CYP3A4 on the VDR‐induction ability of 1,25(OH)_2_D_3_ in C2C12 cells, we pretreated HepG2 cells with 4NQO. The addition of physiological levels (0.1 nM) of 1,25(OH)_2_D_3_ to the HepG2 cell culture medium failed to increase VDR levels in C2C12 cells, and the induction ability of 1 nM of 1,25(OH)_2_D_3_ was partially reduced. Following pretreatment of HepG2 cells with PF (CYP3A4 specific inhibitor), the VDR levels in C2C12 cells were fully restored by 0.1 and 1 nM concentrations of 1,25(OH)_2_D_3_ (Figure 7E). In our liver RNA‐seq results, we observed a significant increase in the stress‐responsive transcription factor *Atf3* in SKO mice, which was validated by qPCR with consistent results and showed a positive correlation with *Cyp3a41* (*R* ^2^ = 0.7135) (Figure S5A,B). In vitro induction of HepG2 cells with 4NQO revealed increased *ATF3* expression (Figure S5C). Using the JASPAR database, we identified binding domains between ATF3 and the CYP3A4 promoter (Figure S5D). Subsequent silencing of ATF3 resulted in reduced 4NQO‐induced *CYP3A4* expression (Figure S5E,F). Dual‐luciferase reporter gene experiments demonstrated that overexpression of ATF3 enhanced *CYP3A4* promoter expression, indicating direct transcriptional regulation of *CYP3A4* expression by ATF3 (Figure 7F).

The findings above show a decrease in active VD levels in both male and female SKO mice. Additionally, the physical activity levels of SKO mice do not significantly differ from those of SWT mice, thereby making inactivity an unlikely factor in the sarcopenia in the SKO mice [22]. Therefore, VD levels and physical activity levels are not factors influencing the sex‐specific effects of VD in SKO mice.

### 3.10 VD and Testosterone Have an Interactive Effect on Grip Strength in Adults

**FIGURE 8:**
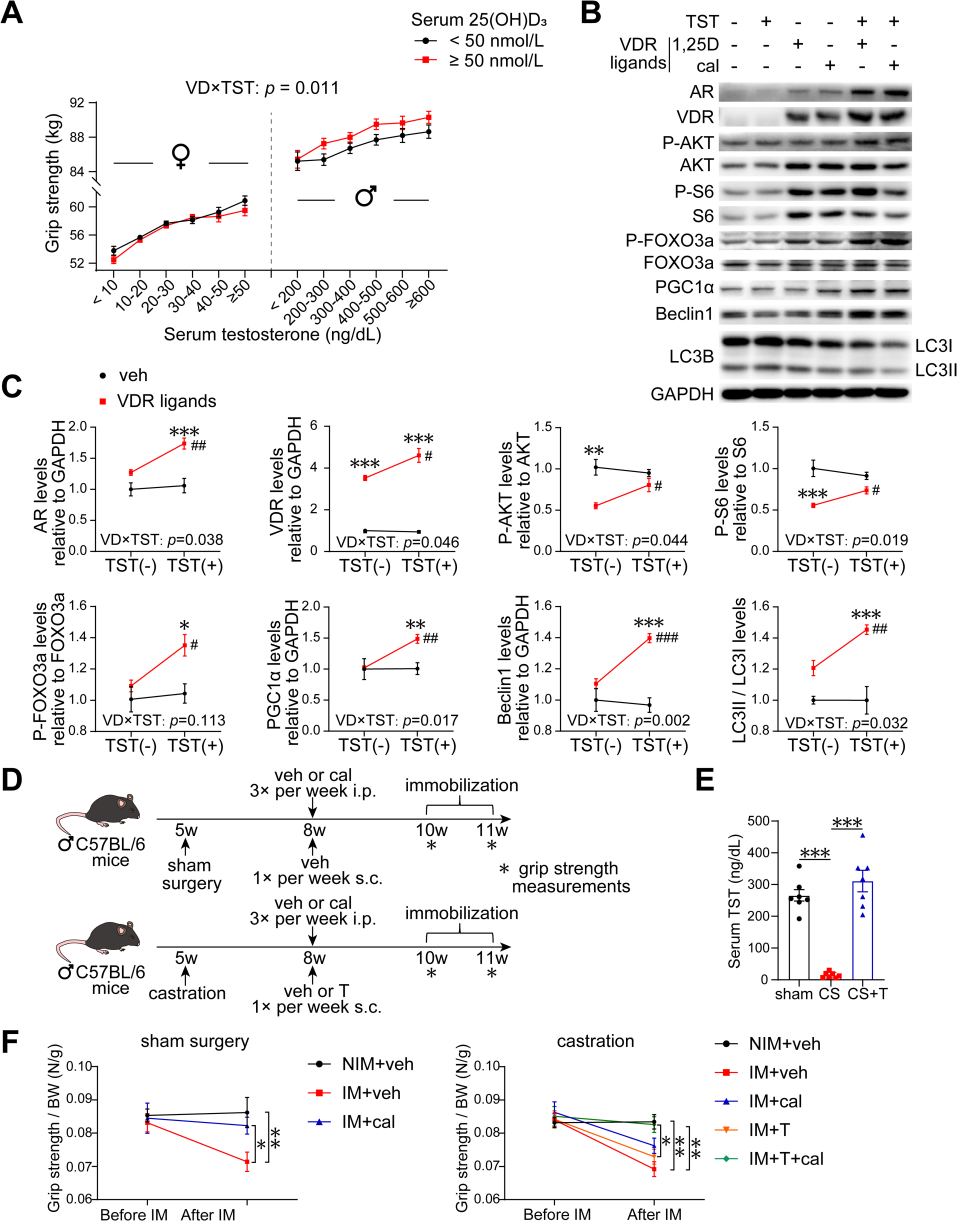
Testosterone enhances muscle strength and molecular response to vitamin D. (A) Diagram of the interactive effect of serum 25(OH)D_3_ level and testosterone on grip strength in male and female adults. Predicted grip strength was adjusted by age, race, body mass index, physical activity, education level, serum calcium and smoking status. (B,C) Representative western blotting results showing the analyses of AR, VDR, P‐AKT, total AKT, P‐S6, total S6, P‐FOXO3a, total FOXO3a, PGC1α, Beclin1 and LC3B (*n* = 3 biological replicates per experimental group). *VDR ligands vs. vehicle; #TST (+) vs. TST (−). (D–F) Experimental scheme (*n* = 6–10) (D). Serum testosterone levels in castrated, sham‐operated and castrated mice supplemented with TSTE (E). Levels of grip strength in mice from the sham surgery and castration groups after intervention (F). Data are represented as mean ± SEM. #,*p < 0.05, ##,**p < 0.01 and ###,***p < 0.001 by one‐way or two‐way ANOVA (two‐tailed) with Tukey's post hoc for multiple comparisons. 1,25D, 1,25(OH)_2_D_3_; cal, calcipotriol; IM, immobilization; NIM, non‐immobilization; T or TSTE, testosterone enanthate; TST, testosterone; VD × TST, the interactive effect of VDR ligands and testosterone; veh, vehicle.

Sex differences in the response of human skeletal muscle to exercise and training, with sex hormones being one of the main contributing factors. The resistance exercise‐induced testosterone response promotes muscle protein synthesis activation in males [23]. To investigate the potential factors contributing to sex‐related variations in the effects of VD on muscle function, we conducted a cross‐sectional study utilizing data from the NHANES database. Participant characteristics, stratified by serum 25(OH)D_3_ levels and sex, are presented in Tables S1 and S2, respectively. Individuals with VD deficiency (< 50 nmol/L) exhibited a higher prevalence among females and demonstrated lower levels of testosterone, grip strength and physical activity in comparison to those without VD deficiency (≥ 50 nmol/L). Serum VD and testosterone had an interactive effect on grip strength (Table S3). A positive correlation between VD and grip strength was observed when testosterone levels exceeded 300 ng/dL (Table S4). A diagram of the interactive effect of VD and testosterone level on grip strength in male and female adults is shown in Figure 8A.

### 3.11 Calcipotriol Increases the Expression of Androgen Receptor in the Gastrocnemius Muscle of Male SKO Mice

Testosterone primarily exerts its effects through the androgen receptor (AR). Given the interaction between testosterone and VD on grip strength, we tested whether calcipotriol affects the expression of the AR in mice as well as serum testosterone levels. Calcipotriol increased the protein levels of AR in male SKO mice and VDR levels in male SWT mice, but it did not significantly affect the mRNA levels (Figure S6A–D). Male mice exhibited markedly higher testosterone levels than female mice, and in males but not in females, the SKO genotype had higher testosterone levels than the SWT. However, calcipotriol did not exhibit significant impacts on testosterone levels in either genotype of both sexes (Figure S6E). Nevertheless, no significant disparity was observed in AR and VDR expression levels between male and female SWT mice (Figure S6F).

### 3.12 Testosterone Enhances the Molecular Response to VD In Vitro

C2C12 cells were induced to differentiate by 2% horse serum with either VDR ligands, testosterone or a combination of both, aiming to investigate potential interactions between VD and testosterone. The results showed that VDR ligands and testosterone exhibit an interactive effect on the VDR signalling molecules. Treatment with testosterone alone had no significant effect on the expressions of the concerned molecules in C2C12 cells. However, testosterone enhanced the up‐regulatory effect of VDR ligands on AR, VDR, P‐AKT, S6, P‐FOXO3a, PGC1α, LC3, and Beclin1, as well as the levels of *Vdr* mRNA (Figures 8B,C and S7A). Additionally, testosterone enhances the up‐regulation of *Mfy5* and *Myf6* induced by VDR ligands but does not significantly enhance the effects on *Myod1* and *Myog*, nor does it promote the hypertrophic effects of VDR ligands on myotubes (Figure S7B,C).

### 3.13 Testosterone Enhances the Ability of Calcipotriol to Improve Grip Strength in Immobilized Mice

Finally, male wild‐type castrated or sham‐operated mice were utilized, and they were administered calcipotriol, testosterone enanthate (TSTE) or a combination of both to investigate whether testosterone enhanced the effect of VD on grip strength in immobilized mice (Figure 8D). Castrated mice showed decreased levels of serum testosterone, which were restored to normal levels after TSTE supplementation (Figure 8E). Castrated mice exhibited reduced body weight, which increased after TSTE supplementation, whereas immobilization caused a decrease in body weight (Figure S7D,E).

Following adjustment for body weight, it was found that in both the sham‐operated and castrated groups, immobilization resulted in a significant decrease in gastrocnemius muscle mass. However, no improvement was observed after treatment with calcipotriol, TSTE or a combination of both (Figure S7F,G). Regarding grip strength in the sham‐operated group, immobilization led to a decrease, which was significantly inhibited by calcipotriol treatment (Figure 8F). In the castrated group, immobilization reduced grip strength, and although treatment with TSTE or calcipotriol alone resulted in a slight, non‐significant increase, their combined effect restored grip strength to the level of non‐immobilized mice, exhibiting a significant increase compared to TSTE treatment alone (Figure 8F).

## 4 Discussion

Sarcopenia is a progressive and systemic disease characterized by a progressive decline in skeletal muscle mass, reduced muscle strength and/or impaired physical performance [S9]. Grip strength is a superior predictor compared to muscle mass for adverse outcomes, including diminished physical performance and increased risk of falls [24]. Therefore, prioritizing the prevention of muscle strength loss becomes crucial. Our findings demonstrate that calcipotriol effectively mitigated the decline in grip strength in male SKO mice and restored grip strength to levels comparable to those of the SWT mice, although no significant effect on muscle mass was observed. Previous studies have indicated that muscle strength is influenced by various factors, such as changes in muscle fibre type, inflammation, mitochondrial function and neuromuscular transmission [25]. Calcipotriol selectively induced an increase in the number of MHC2b‐type myofibres with a CSA ranging from 1000 to 1400 μm^2^ in SKO mice. Additionally, it enhanced protein expression in the range of 35–100 kDa. Muscle‐specific overexpression of VDR increases protein synthesis [9], but this selectivity has not been reported.

TNFα, IL‐1β and IL‐6 promote protein ubiquitination and degradation, inhibit protein synthesis and also stimulate satellite cell proliferation and muscle regeneration [26, S10]. Our results demonstrated that calcipotriol reduced inflammation in the gastrocnemius muscle and promoted the expression of muscle differentiation marker genes. The muscle‐specific E3 ubiquitin ligases TRIM63 and FBXO32 attach ubiquitin to the protein substrate, and the resulting complex is recognized by the 26S proteasome, which degrades the substrate into peptides [27]. Mitochondrial dysfunction is thought to play a major role in muscle atrophy [28]. Enhancement of mitochondrial function restores muscle mass and function in sarcopenia [29]. Calcipotriol enhances mitochondrial biogenesis, restores ATP production capacity and promotes autophagy to remove damaged mitochondria. Additionally, previous studies have found that myocyte‐specific *Vdr* knockout in muscle cells may lead to decreased muscle strength due to alterations in contraction‐relaxation mechanisms via changes in the calcium handling apparatus [8]. However, our RNA‐seq analysis did not indicate any effects of SKO or calcipotriol on the calcium handling signalling pathway.

In this study, the beneficial effects of calcipotriol on muscle strength were observed only in male SKO mice, with no observed effects in females. Basal VD levels and physical activity levels are interfering factors influencing the effect of VD on muscle function [11, 12]. Both male and female SKO mice exhibited decreased levels of active VD. Additionally, the physical activity levels of SKO mice do not significantly differ from those of SWT mice [22]. Prior research has indicated that VD may elevate serum testosterone levels in D‐galactose‐induced aged rats [30]. However, our results showed that calcipotriol had no significant effect on serum testosterone levels but increased the level of AR in the gastrocnemius muscle of male SKO mice. Subsequent experiments in male castrated mice and C2C12 cells demonstrated that testosterone enhanced the grip strength and molecular responses to the VD. VD levels have a weaker effect on grip strength in older adults compared to younger adults [31]. In conjunction with our experimental findings, this phenomenon may be attributed to lower testosterone levels in older adults.

The interaction between VD and testosterone is not limited to muscle cells. Previous studies have indicated that the antiproliferative effect of 1,25(OH)_2_D_3_ on androgen‐dependent prostate cancer cells LNCaP is dependent on androgens, but the effect is unaffected by androgens in androgen‐independent prostate cancer cells C4‐2 [32]. AR and VDR both belong to the steroid receptor superfamily of ligand‐inducible transcription factors. The interaction between VD and testosterone in LNCaP cells might be attributed to the shared transcription targets of AR and VDR [32]. Our findings show that calcipotriol, rather than testosterone, elevated the expression levels of AR and VDR, whereas testosterone notably amplified this effect. This study identified VD increasing protein synthesis and mitochondrial biogenesis, reducing inflammation and muscle atrogenes. Testosterone enhances muscle mass and function by increasing muscle protein synthesis and improving mitochondrial function, as well as inhibiting protein degradation [33]. Thus, the potentiation of VD by testosterone on muscle strength and molecular response may be accomplished through the shared targets of AR and VDR, warranting further investigation.

Recent studies have indicated that participants with non‐alcoholic fatty liver disease (NAFLD) are at a higher risk of sarcopenia [34, S11]. Various hypotheses exist, such as insulin resistance, inflammation, hyperammonemia and VD deficiency [35]. Liver‐related VD deficiency primarily arises from impaired liver function, resulting in decreased 25‐hydroxylation of VD_3_ and subsequent reduction in serum 25(OH)D_3_ levels [36]. Additionally, liver CYP3A4 enzymes are responsible for metabolizing 1,25(OH)_2_D_3_ into inactive metabolites [4]. This study indicated that elevated levels of ROS were primarily responsible for the up‐regulation of CYP3A4 expression. ATF3 is a stress‐activated transcription factor that remains relatively stable under normal physiological conditions. However, it rapidly changes in response to internal or external disruptions, such as inflammatory reactions, oxidative stress, DNA damage or endoplasmic reticulum stress [37]. Our research found that ATF3 directly regulated the expression of CYP3A4 by activating its gene promotor.

This study revealed a decline in serum 1,25(OH)_2_D_3_ levels in SKO mice at 18 and 28 weeks, preceding the decrease in serum 25(OH)D_3_. Prior studies have demonstrated an increase in *Cyp3a* during the initial stages of hepatic steatosis induced by a high‐fat diet [38]. Conversely, patients with cirrhosis and hepatocellular carcinoma exhibit diminished hepatic CYP3A4 enzyme activity [39]. The results suggest that relying solely on serum 25(OH)D_3_ levels for diagnosing VD deficiency may not be timely for certain liver disease patients. It holds important value to detect VD deficiency promptly through the measurement of serum 1,25(OH)_2_D_3_ or the development of methods to detect VD metabolites of CYP3A4.

In conclusion, this study reveals that the effect of VD on muscle strength is influenced by sex. Testosterone is the sex‐selective factor influencing the action of VD, which can enhance muscle strength and molecular response to VD. Therefore, this study highlights the importance of considering testosterone levels when utilizing VD to enhance muscle strength.

## Conflicts of Interest

The authors declare no conflicts of interest.

## Supporting information


**Figure S1** Calcipotriol increased MHC2b in gastrocnemius muscle of male SKO mice. (A,B) mRNA levels of gastrocnemius MHC isoform genes in male (A) and female (B) mice. (C,D) Mean myofibre cross‐sectional areas (C) and myofibre count (D) in H&E stains. (E,F) Mean myofibre cross‐sectional areas (K) and myofibre count (L) in MHC2b stains. Data are represented as mean ± SEM. *,†*p* < 0.05, **,††*p* < 0.01 and ***,†††*p* < 0.001 by two‐way ANOVA (two‐tailed) with Tukey’s post hoc for multiple comparisons. †Main effect of SWT vs. SKO. cal, calcipotriol; veh, vehicle.
**Figure S2** RNA sequencing analysis and myofibre differentiation genes in the gastrocnemius of mice. (A) The number of significantly differentially expressed genes by RNA sequencing of gastrocnemius (*n* = 3). (B) GSEA showing proteasome pathways up‐regulated at male SKO compared to SWT mice. Heat maps show the expression of the core genes that contribute to pathway enrichment (*n* = 3). (C,D) mRNA levels of gastrocnemius myofibre differentiation genes measured by qPCR of male (C) and female (D) mice (*n* = 6–8). Data are represented as mean ± SEM. *,†*p* < 0.05, **p < 0.01 and ***,†††*p* < 0.001 by two‐way ANOVA (two‐tailed) with Tukey’s post hoc for multiple comparisons. †Main effect of SWT vs. SKO. cal, calcipotriol; GSEA, Gene Set Enrichment Analysis; NES, normalized enrichment score; veh, vehicle.
**Figure S3** ROS induces overexpression of CYP3A4 in hepatocytes. (A,B) mRNA levels of vitamin D metabolism‐related genes in the kidneys (A) and liver (B) in male mice (*n* = 3–7). (C,D) C57BL/6 male mice were treated with 4NQO (250 μg/kg body weight) or vehicle once daily for 2 days (*n* = 7 each). mRNA levels of genes related to vitamin D metabolism in the liver (C) and kidneys (D) measured by qPCR. (E) Expression levels of *CYP3A4* mRNA at 6, 24 and 48 h in HepG2 cells treated with 4NQO (1 μM) or vehicle (*n* = 3). (F) mRNA levels of *CYP3A4* in HepG2 cells treated with 4NQO (1 μM), Tempol (SOD mimetic agent) (100 μM) or vehicle (*n* = 3). Data are represented as mean ± SEM. *p < 0.05, **p < 0.01 and ***p < 0.001 by one‐way or two‐way ANOVA (two‐tailed) with Tukey’s post hoc for multiple comparisons. veh, vehicle; cal, calcipotriol.
**Figure S4** Vitamin D metabolism parameters effect by VDR ligand. (A–D) The level of serum 25(OH)D_3_ (A), 1,25(OH)_2_D (B), calcium (C) and phosphorus (D). (E,F) Expression level of genes related to vitamin D metabolism in the liver (E) and kidneys (F) measured by qPCR. Data are represented as mean ± SEM. *,†*p* < 0.05, **p < 0.01 and ***,†††*p* < 0.001 by two‐way ANOVA (two‐tailed) with Tukey’s post hoc for multiple comparisons. †Main effect of SWT vs. SKO. cal, calcipotriol; veh, vehicle.
**Figure S5** ROS induces ATF3 transcription to regulate CYP3A4 expression. (A,B) Expression level of *Atf3* in the liver of male SWT and SKO mice at 4, 8, 18 and 28 weeks (A). Correlations with regression lines to examine the relationship between liver *Cyp3a41* and *Atf3* mRNA levels (B). The mRNA level 2^−ΔΔCt^ was log‐transformed for analysis (*n* for each group same as Figure 1A). (C) Expression levels of *ATF3* at 2, 6, 24 and 48 h in HepG2 cells treated with 4NQO (1 μM) or vehicle (*n* = 3). (D) Schematic diagram of the putative ATF3 binding site in the proximal region of the human (h) CYP3A4 and mouse (m) Cyp3a41 promoter. (E) Expression levels of ATF3 measured by immunoblotting. HepG2 cells were transfected with ATF3 shRNA or control shRNA, and HEK 293T cells were transfected with ATF3 overexpression plasmid or control pcDNA3.1 plasmid for 48 h. (F) mRNA levels of *CYP3A4* in HepG2 cells pretreated with ATF3 shRNA or control shRNA, then treated with 4NQO (1 μM) or vehicle for 24 h (*n* = 3). Data are represented as mean ± SEM. *p < 0.05 and ***p < 0.001 by one‐way or two‐way ANOVA (two‐tailed) with Tukey’s post hoc for multiple comparisons, or simple linear regression. cal, calcipotriol; veh, vehicle.
**Figure S6** The effect of calcipotriol on AR, VDR and testosterone levels. (A,B) Expression level of gastrocnemius *Ar* and *Vdr* by qPCR of SWT and SKO mice treated calcipotriol or vehicle (*n* = 6–8). (C,D) Representative western blotting results showing the analysis of AR and VDR in gastrocnemius of male (C) and female (D) mice (*n* = 6 biological replicates per experimental group). (E) The serum testosterone level of male and female mice (*n* = 6–8). (F) Western blotting results showing AR and VDR in gastrocnemius of SWT male and female mice (*n* = 4 biological replicates per experimental group). Data are represented as mean ± SEM. *,†*p* < 0.05, **p < 0.01 and ***,†††*p* < 0.001 by two‐way ANOVA (two‐tailed) with Tukey’s post hoc for multiple comparisons or by Student’s unpaired two‐tailed *t*‐test. †Main effect of SWT vs. SKO. cal, calcipotriol; veh, vehicle.
**Figure S7** Testosterone influences the effects of vitamin D on muscle. (A,B) Expression level of *Ar*, *Vdr* (A) and myofibre differentiation genes (B) measured by qPCR. (C) Representative images of C2C12 myotubes, and the diameter of myotubes was measured. Scale bars, 100 μm. Differentiation of C2C12 cells was induced by 2% horse serum plus every 2 days’ treatment of testosterone, VDR ligands, or vehicle for 8 days (*n* = 3). [*VDR ligands vs. vehicle, #TST (+) vs. TST (−)]. (D–G) Levels of body weight (B,C) and gastrocnemius muscle mass (D,E) in mice from the sham surgery and castration groups after intervention (*n* = 6–10). [$IM + veh vs. IM + T, #IM + veh vs. NIM + veh]. Data are represented as mean ± SEM. *p < 0.05, ##*p* < 0.01 and $$$,###,***p < 0.001 by two‐way ANOVA (two‐tailed) with Tukey’s post hoc for multiple comparisons. cal, calcipotriol; IM, immobilization; NIM, non‐immobilization; T or TSTE, testosterone enanthate; TST, testosterone; veh, vehicle.
**Table S1** Descriptive characteristics of the study population stratified by serum 25(OH)D_3_ level.
**Table S2** Descriptive characteristics of the study population stratified by sex.
**Table S3** Linear regression analysis of grip strength in adults.
**Table S4** Linear regression analysis of grip strength stratified by testosterone in adults.
**Table S5** qPCR primer sequences.
**Table S6** List of antibodies used in this study.
