# Supplementary material for: The Effects of Vitamin D on Muscle Strength Are Influenced by Testosterone Levels
Source: J Cachexia Sarcopenia Muscle. 2025 Feb 16;16(1):e13733. doi: 10.1002/jcsm.13733 (PMC11830628; doi:10.1002/jcsm.13733)
Supplement: Supplementary file 1 — Figure S1 Calcipotriol increased MHC2b in gastrocnemius muscle of male SKO mice. (A,B) mRNA levels of gastrocnemius MHC isoform genes in male (A) and female (B) mice. (C,D) Mean myofibre cross‐sectional areas (C) and myofibre count (D) in H&E stains. (E,F) Mean myofibre cross‐sectional areas (K) and myofibre count (L) in MHC2b stains. Data are represented as mean ± SEM. *,†p < 0.05, **,††p < 0.01 and ***,†††p < 0.001 by two‐way ANOVA (two‐tailed) with Tukey’s post hoc for multiple comparisons. †Main effect of SWT vs. SKO. cal, calcipotriol; veh, vehicle. Figure S2 RNA sequencing analysis and myofibre differentiation genes in the gastrocnemius of mice. (A) The number of significantly differentially expressed genes by RNA sequencing of gastrocnemius (n = 3). (B) GSEA showing proteasome pathways up‐regulated at male SKO compared to SWT mice. Heat maps show the expression of the core genes that contribute to pathway enrichment (n = 3). (C,D) mRNA levels of gastrocnemius myofibre differentiation genes measured by qPCR of male (C) and female (D) mice (n = 6–8). Data are represented as mean ± SEM. *,†p < 0.05, **p < 0.01 and ***,†††p < 0.001 by two‐way ANOVA (two‐tailed) with Tukey’s post hoc for multiple comparisons. †Main effect of SWT vs. SKO. cal, calcipotriol; GSEA, Gene Set Enrichment Analysis; NES, normalized enrichment score; veh, vehicle. Figure S3 ROS induces overexpression of CYP3A4 in hepatocytes. (A,B) mRNA levels of vitamin D metabolism‐related genes in the kidneys (A) and liver (B) in male mice (n = 3–7). (C,D) C57BL/6 male mice were treated with 4NQO (250 μg/kg body weight) or vehicle once daily for 2 days (n = 7 each). mRNA levels of genes related to vitamin D metabolism in the liver (C) and kidneys (D) measured by qPCR. (E) Expression levels of CYP3A4 mRNA at 6, 24 and 48 h in HepG2 cells treated with 4NQO (1 μM) or vehicle (n = 3). (F) mRNA levels of CYP3A4 in HepG2 cells treated with 4NQO (1 μM), Tempol (SOD mimetic agent) (100 μM) or vehicle (n = 3). D [file JCSM-16-e13733-s001.docx]

The effects of vitamin D on muscle strength are influenced by testosterone levels

Aolin Yang^1,2^, Qingqing Lv^1^, Ziyu Han^1^, Shimiao Dai^1^, Yao Li^1^, Mengru Hao^1^, Ruirui Yu^1^, Junying Zhu^1^, Chenggang Yang^1^, Zhan Shi^1^, Ji-Chang Zhou^1^^,3,4^*

Aolin Yang and Qingqing Lv contributed equally to this work.

* Correspondence: zhoujch8@mail.sysu.edu.cn.

**Supplemental Methods**

**Supplemental Figures and Tables**

**Supplemental References**

## Supplemental Methods

**Cells**

Cells were grown at 37°C in 5% CO_2_, and cultured in DMEM with 4.5 g/L glucose (C11885500BT, GIBCO, Grand Island, NY, USA) supplemented with 10% FBS (13011-8611, Every Green, Zhejiang, China) and 1% penicillin / streptomycin (SV30010, HyClone, South Logan, UT, USA). For C2C12 cells differentiation, the serum was changed to 2% horse serum (SBJ-SE-HO014, SenBeiJia, Nanjing, China).

## Genotyping

Genotyping of the SKO mice was performed with alkaline extracted tail genomic DNA by qPCR. Different genotypes showed distinct melting temperature (Tm) values. The genotyping oligonucleotide primers used are shown in Table S6.

## Surgical Castration

The mice were anesthetized with isoflurane and placed in a supine position on the surgical table. The area below the abdomen of the mouse was shaved, followed by disinfection using iodine tincture and 75% alcohol-soaked cotton balls. The testes were pushed into the scrotum, and a 0.5 cm incision was made along the midline of the scrotum, followed by blunt dissection. Forceps were used to remove the fat, revealing the elliptical testis. The testis was carefully ligated at the upper and lower ends with absorbable sutures, and then the testis and epididymis were removed using ophthalmic scissors, with the remaining tissue returned to the abdominal cavity. After the removal of both testes, the skin was sutured and disinfected. Postoperatively, the mice were placed on a 37°C heating pad for recovery, and once awake, they were returned to their cages. In the sham surgery group, the testes were pulled out and then returned without ligation and removal.

**Primary hepatocyte isolation**

Primary hepatocyte isolation was performed using a two-step collagenase perfusion technique. In the first step, the inferior vena cava is cannulated after giving anesthesia and the liver is perfused with 80 mL HBSS buffer without Ca^2+^ and Mg^2+^ and containing 0.5 mM EDTA to chelate calcium and wash out blood. In the second step, the enzyme collagenase is introduced into the liver lobes by 100 mL DMEM with 100 U/mL collagenase IV (40510ES76, Yeasen, Shanghai, China) and 5 mM HEPES (V900477, Sigma, St. Louis, MO, USA), and the perfused collagenase dissociates the extracellular matrix of liver cells. Finally, the liver is separated from the mouse, and liver cells are purified by centrifugation for 3 min at 50 × g, 4 °C, and washed three times by DMEM.

## Serum measurements

Serum 1,25(OH)_2_D_3_ and testosterone levels were determined using 1,25-Dihydroxyvitamin D3 (DHVD3) Enzime-linked Immunosorbent Assay (ELISA) Kit (CEA467Ge, cloud-clone, Wuhan, China) and testosterone ELISA Kit (PT872, Beyotime, Shanghai, China). Serum Calcium and Phosphate (C004-2-1, C006-1-1, Jiancheng, Nanjing, China) were measured following the manufacturer’s instructions. Serum 25(OH)D_3_ was determined by liquid chromatography-tandem mass spectrometry.

**Transcriptomic analysis**

RNA was extracted from the liver or gastrocnemius with Total RNA Extraction Reagent. The RNA submitted to Genedenovo Biotechnology Co., Ltd (Guangzhou, China), and RNA quality was assessed on an Agilent 2100 Bioanalyzer (Agilent Technologies, Palo Alto, CA, USA). The enriched mRNA was fragmented into short fragments using fragmentation buffer and reversely transcribed into cDNA by using NEBNext Ultra RNA Library Prep Kit for Illumina (NEB#7530, New England Biolabs). The resulting cDNA library was sequenced using Illumina Novaseq6000. Reads were aligned to the mouse (*Mus musculus*) with genome-build Ensembl_release107 and expected counts were generated with Ensembl gene IDs.

RNA differential expression analysis was performed by DESeq2 software between two different groups. The genes with the parameter of false discovery rate (FDR) < 0.05 and |log2FoldChange| > 1 were considered DEGs. We performed gene set enrichment analysis using software GSEA to identify whether a set of genes in specific GO terms/KEGG pathways/Reactome pathways showed significant differences in two groups. Briefly, we inputted the gene expression matrix and ranked genes by SignaltoNoise normalization method. Enrichment scores and *p* value were calculated in default parameters.

**Quantitative PCR**

The mice tissues and cells were extracted with Total RNA Extraction Reagent (10606ES60, Yeasen, Shanghai, China). Then, 1 μg of RNA was used to generate cDNA (11142ES60, Yeasen). Primers for qPCR were obtained from Sangon Biotech (Shanghai, China). Reactions were run on a LightCycler 480 machine (Roche, Basel, Switzerland) with Sybr Green Master Mix (11184ES08, Yeasen). *Gapdh* levels were used to normalize the specific gene expression levels. Primer sequences can be found in Table S5.

**Histology and image analysis**

**Muscle H&E staining**

Gastrocnemius muscle was dissected and fixed overnight in 4% formaldehyde at 4°C. After dehydration in gradient concentrations of sucrose, the muscle was then embedded with optimal cutting temperature (OCT). The 10 μm slices were cut at midbelly and stained with hematoxylin and eosin (H&E) (G1005, Servicebio, Wuhan, China) following the manufacturer’s instructions.

**Immunohistochemistry staining for MHC isoforms**

Fiber typing was performed by immunohistochemistry of frozen 10 μm cut sections and mounted on glass slides. Air-dried sections were immediately blocked in TBS with 0.1% tween-20 (TBST), 0.1% Triton X-100, and 1% bovine serum albumin (BSA) for 1.5 hours at room temperature and immunostained using antibodies to MHC2b (BF-F3 from DSHB deposited by S. Schiaffino, 1:20) diluted in TBST/1%BSA overnight at 4°C. Secondary antibodies against IgG H&L Alexa488 (ab150113, 1:1000, Abcam, Cambridge, MA, USA) diluted in TBST/1%BSA were applied for 1 hour at room temperature and then nuclei were counterstained with Hoechst 33342.

H&E staining and MHC2b staining images were acquired using KEYENCE BZ-X800 (Japan) all-in-one fluorescence microscope with 10x/0.45 N.A. objectives and individual fields were stitched and analyzed using Keyence Advanced Analysis Software. Cross-sectional area fiber size and count were analyzed with ImageJ (ImageJ, Fiji). Minimum and maximum threshold values were established and watershed was used to automatically segment individual fibers, and a mean gray value for the myofiber was then calculated.

**Silver stain**

Skeletal muscle myosin heavy chain (MHC) composition was determined by distinguishing all four MHC isoforms, MHC2a, MHC2x, MHC2b, and MHC1. Gastrocnemius muscle was homogenized in a sodium dodecyl sulfate (SDS) solution containing 10% w/v SDS, 40 mM dithiothreitol (DTT), 5 mM EDTA, and 0.1 M Tris–HCl buffer, pH 8.0, and added Protease Inhibitor Cocktail (78442, Thermo, Waltham, MA, USA). The samples were diluted in 2× loading buffer [100 mM DTT, 4.0% w/v SDS, 0.16 M Tris–HCl (pH 6.8), 40% v/v glycerol, and 0.2% w/v bromophenol blue] and then heated at 100°C for 5 min. The separating gel consisted of 32% v/v glycerol, 8% w/v acrylamide with a 50:1 ratio of acrylamide to Bis, 0.2 M Tris–HCl (pH 8.8), 0.1 M glycine, 0.4% w/v SDS, 0.1% w/v ammonium persulfate (APS), and 0.05% v/v N,N,N′,N′-tetramethylethylenediamine (TEMED). The stacking gel comprised 32% v/v glycerol, 4% w/v acrylamide-Bis (50:1), 70 mM Tris–HCl (pH 6.8), 4 mM EDTA, 0.4% w/v SDS, 0.1% w/v APS, and 0.05% v/v TEMED. The gel was then run using two different buffers, i.e., the lower running buffer consisting of 0.05 M Tris-base, 75 mM glycine, and 0.05% w/v SDS, and the upper running buffer, which was at 6× the concentration of the lower running buffer and had DTT added (final concentration: 1 mM). The gel underwent electrophoresis at 150 V at 4°C for 20 hours except for the first 1 hour, when the maximum current was limited to 10 mA for stacking gel penetration. A silver stain kit (1610449, BIO-RAD, Hercules, CA, USA) was used to stain the gel and visualize the MHC bands immediately after running. The images were captured by a camera (ILCE-6000, Sony, Tokyo, Japan), and the densitometry analysis was conducted using ImageJ software.

**Western blotting**

Gastrocnemius muscle tissue was placed in radioimmunoprecipitation assay lysis buffer (P0013B, Beyotime, Shanghai, China) and homogenized, then centrifuged at 12000 g for 15 min at 4°C to obtain the protein lysate. The lysate was diluted in 5× loading buffer (P0015L, Beyotime, Shanghai, China), subjected to 8-12% SDS-PAGE, and then transferred onto a polyvinylidene fluoride (PVDF) membrane. After being blocked with 5% BSA in Tris-buffered saline/Tween-20 (TBST) buffer (20 mM Tris, 150 mM NaCl, 0.1% Tween-20, pH 7.5) for 1 hour at room temperature, membranes were incubated with primary antibodies overnight at 4°C, and then incubated with a horseradish peroxidase (HRP)-conjugated secondary antibody for 1 hour at room temperature. Then, protein expression levels were evaluated using an ECL luminescent substrate, and images were captured using a Mini Chemiluminescent Imaging and Analysis System (MiniChemi910, SINSAGE, Beijing, China). Images were quantified using ImageJ software and normalized to GADPH or total protein. For antibodies see the Table S6.

## Dual-luciferase reporter assays

293T cells were plated in a 24-well plate and transfected with firefly luciferase plasmids containing the CYP3A4 promoter sequence and/or ATF3 overexpression plasmids, along with control plasmids. All samples were co-transfected with a renilla luciferase plasmid as a control. After 48 hours post-transfection, the activities of firefly and renilla luciferases were measured using dual Luciferase Reporter Gene Assay Kit (11402ES60, Yeasen, Shanghai, China). The relative fluorescence intensity was calculated based on the ratio of firefly to renilla luminescence data.

## Myotubes diameter measurement

The myotubes were photographed with an optical microscope, the diameter of the myotubes were measured by ImageJ software, and the average diameter of the myotubes was calculated by measuring the maximum diameter of each myotube. Take 5 random culture fields for each sample.

**Quantification and statistical analysis**

The methods for population data analysis are as follows. For descriptive analysis, continuous variables are expressed as the mean ± standard deviation (SD) or median and interquartile range (IQR), and categorical variables are expressed as frequencies (%). Continuous variables were analyzed by Student’s unpaired two-tailed *t*-test, and categorical variables were analyzed by the *χ^2^* test. The associations of serum 25(OH)D_3_ and testosterone with handgrip strength were analyzed by linear regression analysis using two separate models. Model 1 was adjusted for age and BMI; Model 2 was adjusted for the variables included in Model 1 plus race, education level, physical activity, smoking status, and serum calcium level. To detect the interaction of serum 25(OH)D_3_ with testosterone, we added the interaction terms from all linear regression equations. Testosterone and 25(OH)D_3_ were processed using log-transformed and box-cox-transformed variables, respectively. Statistical significance was indicated by a two-tailed α level of 0.05.

## Supplemental Figures and Tables

**Figure S1**


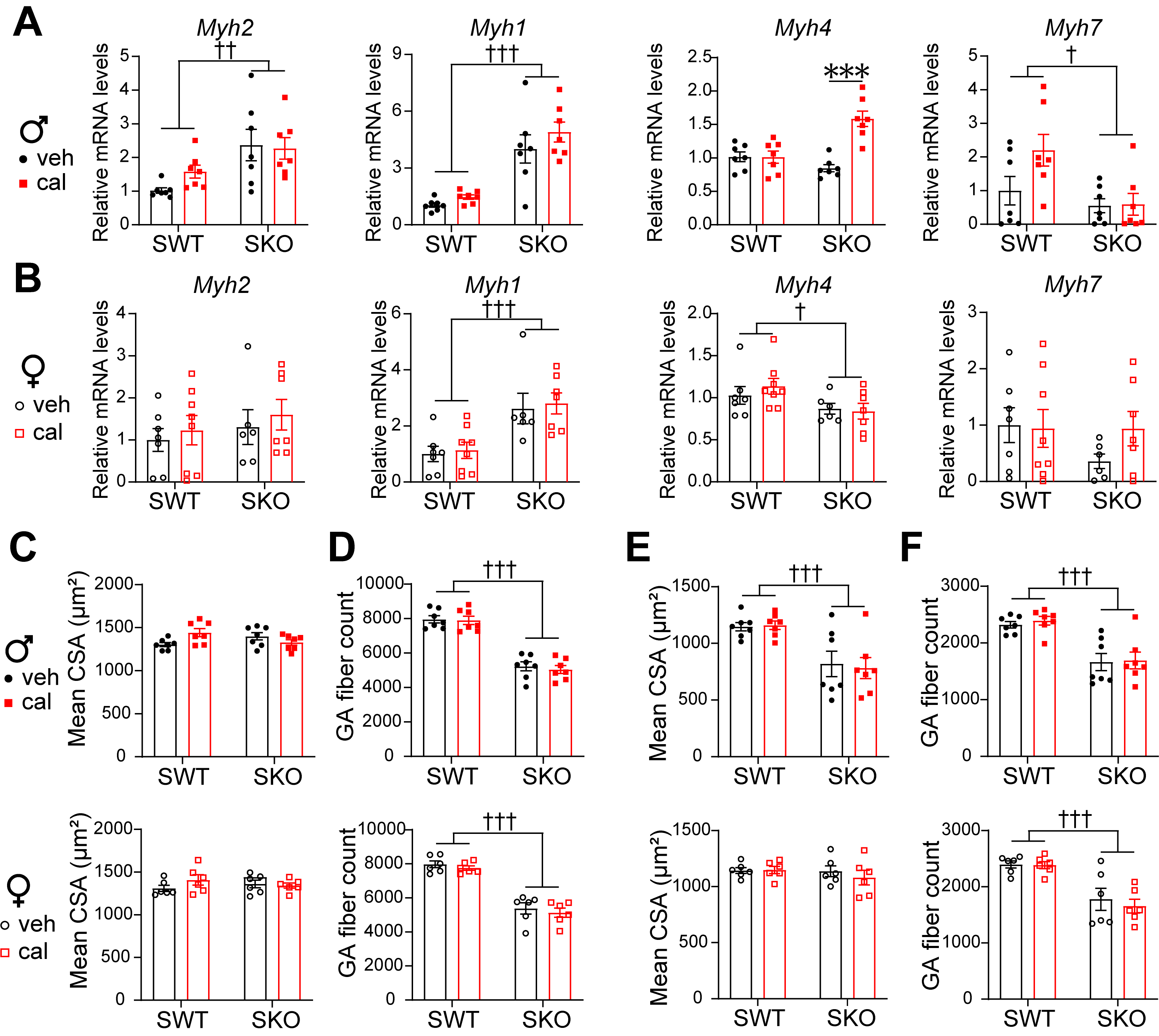


**Figure S1** Calcipotriol increased MHC2b in gastrocnemius muscle of male SKO mice. (A and B) mRNA levels of gastrocnemius MHC isoform genes in male (A) and female (B) mice. (C and D) Mean myofiber cross-sectional areas (C) and myofiber count (D) in H&E stains. (E and F) Mean myofiber cross-sectional areas (K) and myofiber count (L) in in MHC2b stains. Data are represented as mean ± SEM. *, †*p* < 0.05, **, ††*p* < 0.01, and ***, †††*p* < 0.001 by two-way ANOVA (two-tailed) with Tukey’s *post hoc* for multiple comparisons. † Main effect of SWT vs. SKO. veh, vehicle; cal, calcipotriol.

**Figure S2**


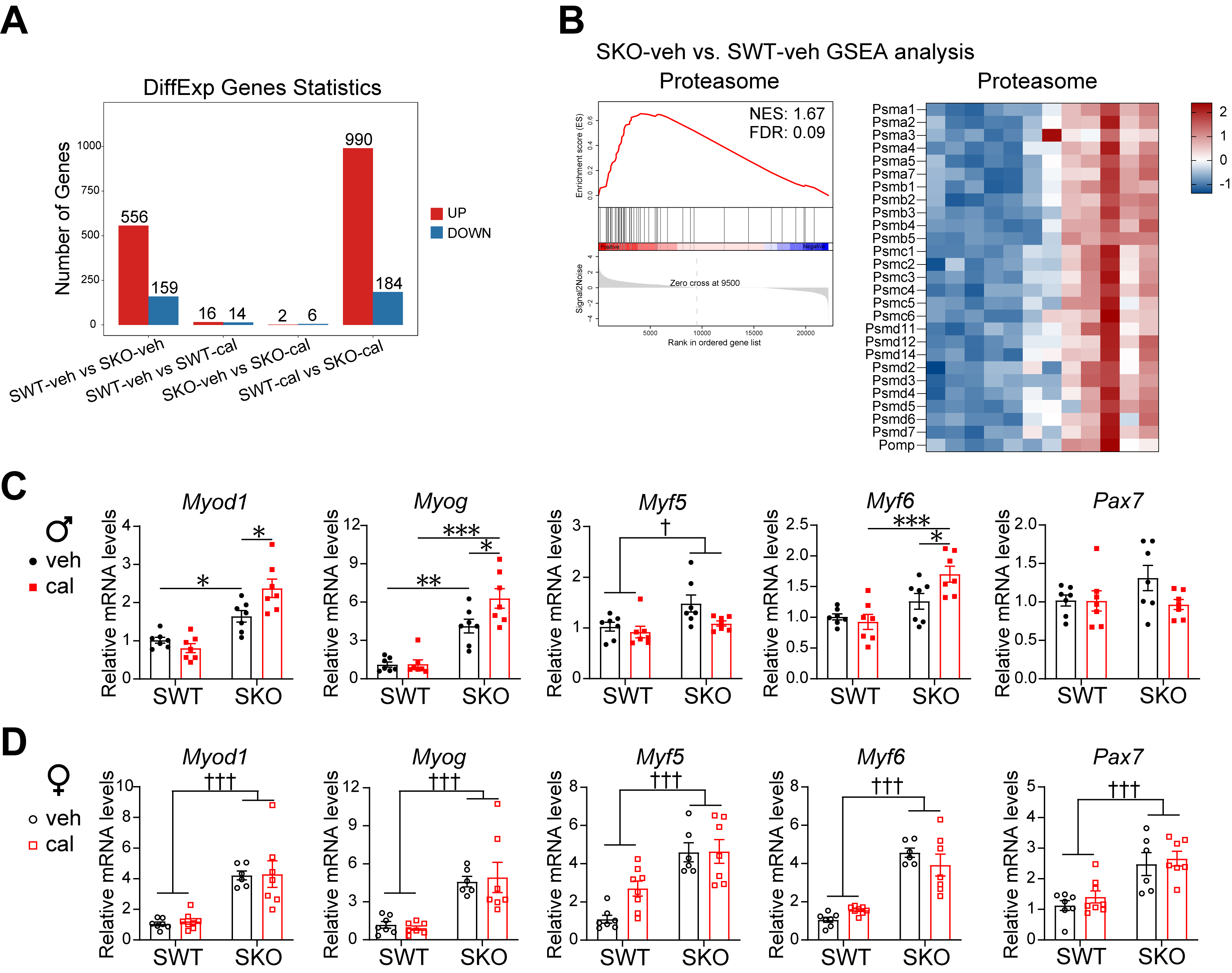


**Figure S2** RNA sequencing analysis and myofiber differentiation genes in the gastrocnemius of mice. (A) The number of significantly differentially expressed genes by RNA sequencing of gastrocnemius (*n* = 3). (B) GSEA showing Proteasome pathways up-regulated at male SKO compared to SWT mice. Heat maps show the expression of the core genes that contribute to pathway enrichment (*n* = 3). (C and D) mRNA levels of gastrocnemius myofiber differentiation genes measured by qPCR of male (C) and female (D) mice (*n* = 6-8). Data are represented as mean ± SEM. *, †*p* < 0.05, ***p* < 0.01, and ***, †††*p* < 0.001 by two-way ANOVA (two-tailed) with Tukey’s *post hoc* for multiple comparisons. † Main effect of SWT vs. SKO. GSEA, Gene Set Enrichment Analysis; NES, normalized enrichment score; veh, vehicle; cal, calcipotriol.

**Figure S3**

**
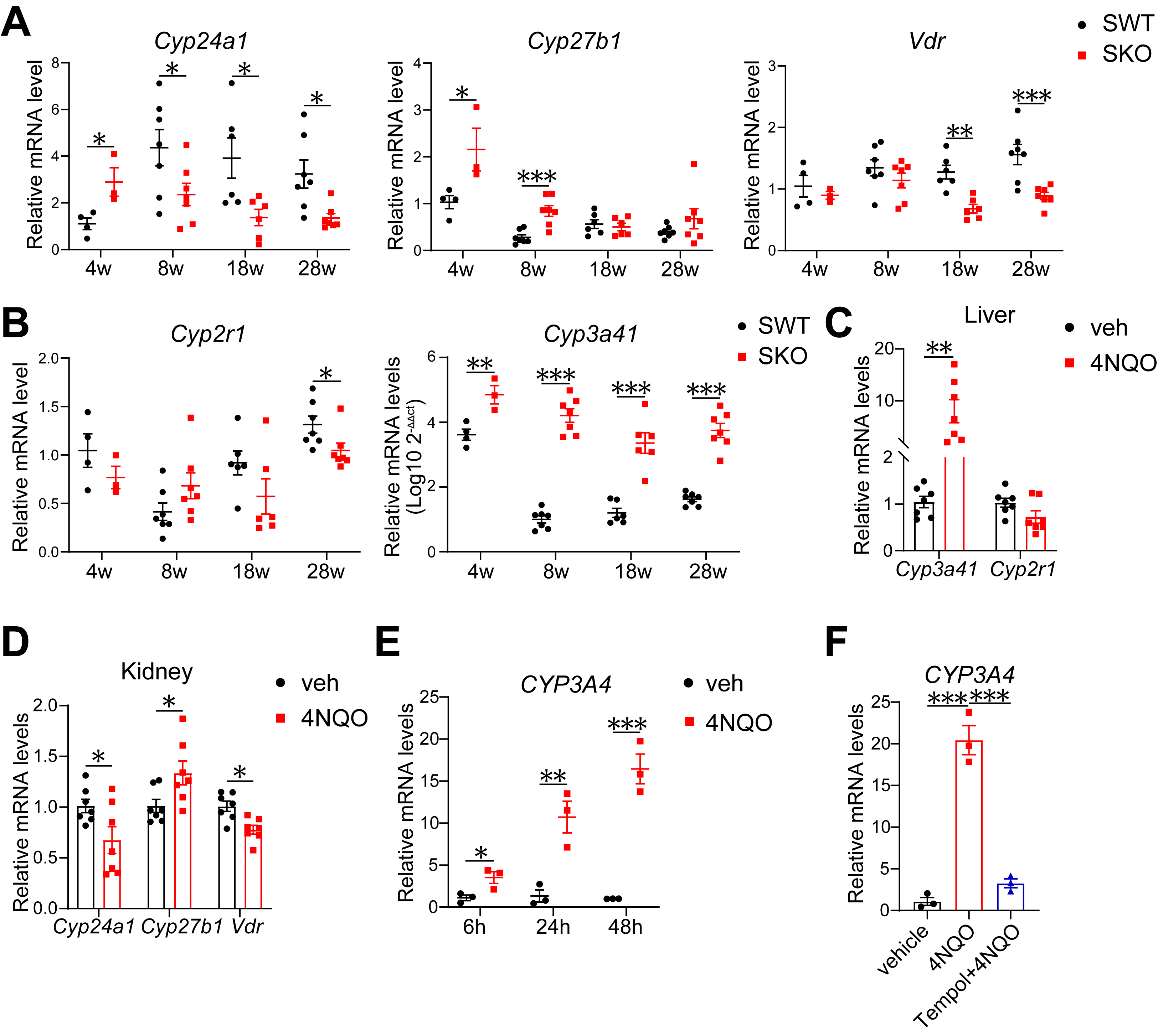
**

**Figure S3** ROS induces overexpression of CYP3A4 in hepatocytes. (A and B) mRNA levels of vitamin D metabolism-related genes in the kidneys (A) and liver (B) in male mice (*n* = 3-7). (C and D) C57BL/6 male mice were treated with 4NQO (250 μg/kg body weight) or vehicle once daily for 2 days (*n* = 7 each). mRNA levels of genes related to vitamin D metabolism in the liver (C) and kidneys (D) measured by qPCR. (E) Expression levels of *CYP3A4* mRNA at 6, 24, and 48 hours in HepG2 cells treated with 4NQO (1 μM) or vehicle (*n* = 3). (F) mRNA levels of *CYP3A4* in HepG2 cells treated with 4NQO (1 μM), Tempol (SOD mimetic agent) (100 μM), or vehicle (*n* = 3). Data are represented as mean ± SEM. **p* < 0.05, ***p* < 0.01, and ****p* < 0.001 by one-way or two-way ANOVA (two-tailed) with Tukey’s *post hoc* for multiple comparisons. veh, vehicle; cal, calcipotriol.

**Figure S4**

**
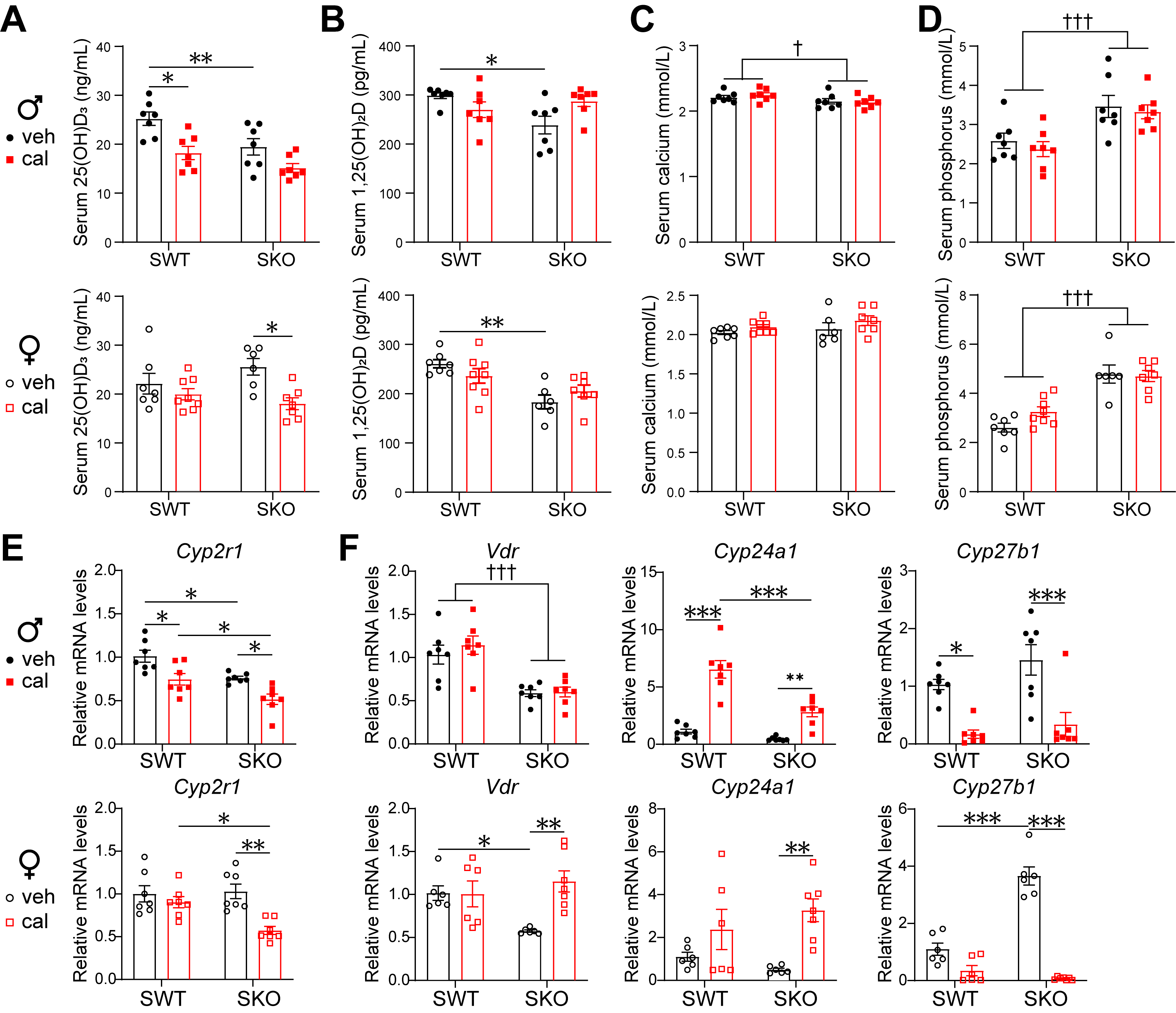
**

**Figure S4. Vitamin D metabolism parameters effect by VDR ligand.** (A-D) The level of serum 25(OH)D_3_ (A), 1,25(OH)_2_D (B), calcium (C), and phosphorus (D). (E and F) Expression level of genes related to vitamin D metabolism in the liver (E) and kidneys (F) measured by qPCR. Data are represented as mean ± SEM. *, †*p* < 0.05, ***p* < 0.01, and ***, †††*p* < 0.001 by two-way ANOVA (two-tailed) with Tukey’s *post hoc* for multiple comparisons. † Main effect of SWT vs. SKO. veh, vehicle; cal, calcipotriol.

**Figure S5**

**
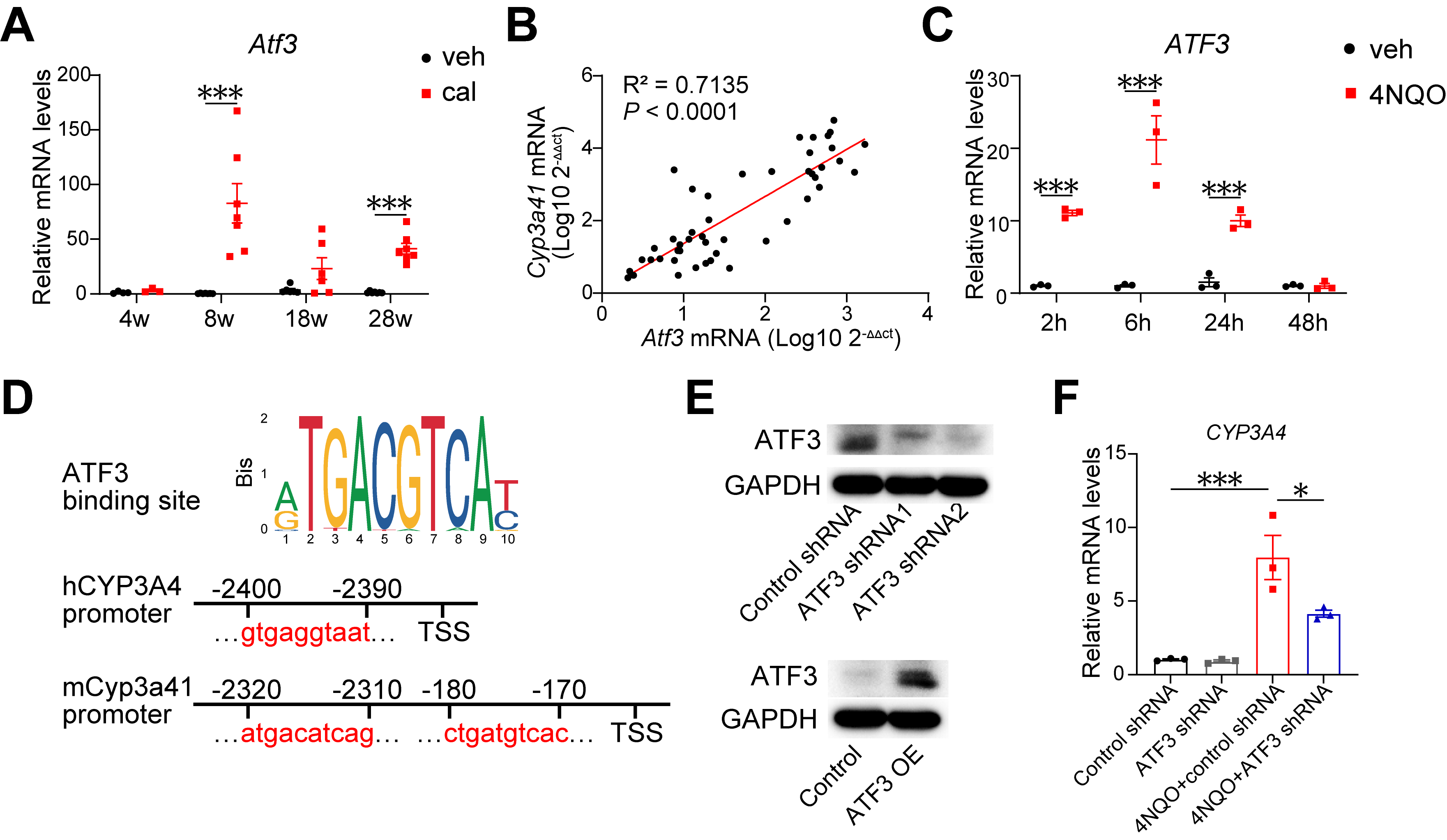
**

**Figure S5.** ROS induces ATF3 transcription to regulate CYP3A4 expression. (A and B) Expression level of *Atf3* in the liver of male SWT and SKO mice at 4, 8, 18, and 28 weeks (A). Correlations with regression lines to examine the relationship between liver *Cyp3a41* and *Atf3* mRNA levels (B). The mRNA level 2^-ΔΔCt^ was log-transformed for analysis (*n* for each group same as Figure 1A). (C) Expression levels of *ATF3* at 2, 6, 24, and 48 hours in HepG2 cells treated with 4NQO (1 μM) or vehicle (*n* = 3). (D) Schematic diagram of the putative ATF3 binding site in the proximal region of the human (h) CYP3A4 and mouse (m) Cyp3a41 promoter. (E) Expression levels of ATF3 measured by immunoblotting. HepG2 cells were transfected with ATF3 shRNA or control shRNA, and HEK 293T cells were transfected with ATF3 overexpression plasmid or control pcDNA3.1 plasmid for 48 hours. (F) mRNA levels of *CYP3A4* in HepG2 cells pre-treated with ATF3 shRNA or control shRNA, then treated with 4NQO (1 μM) or vehicle for 24 hours (*n* = 3). Data are represented as mean ± SEM. **p* < 0.05 and ****p* < 0.001 by one-way or two-way ANOVA (two-tailed) with Tukey’s *post hoc* for multiple comparisons, or simple linear regression. veh, vehicle; cal, calcipotriol.

**Figure S6**

**
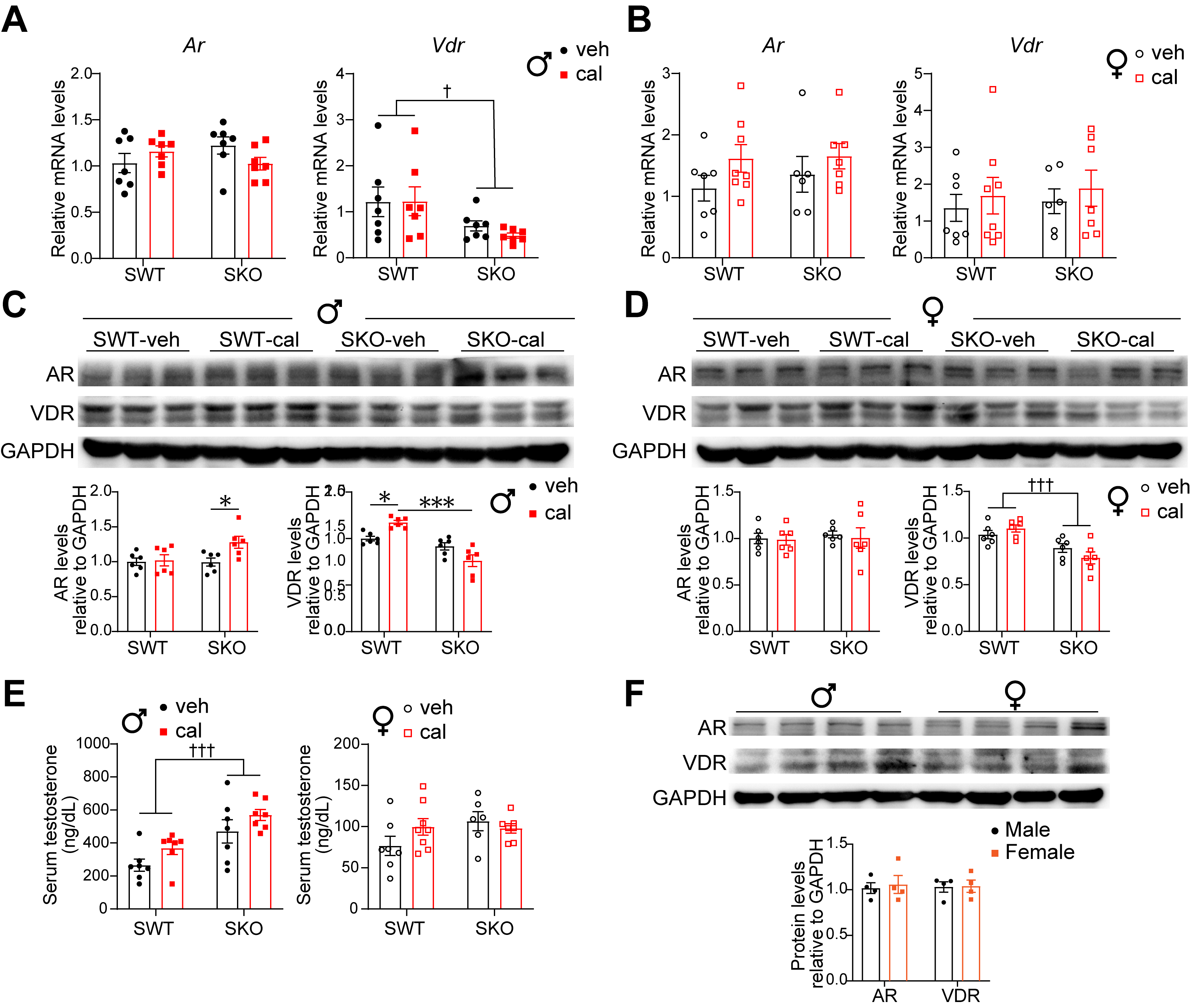
**

**Figure S6** The effect of calcipotriol on AR, VDR, and testosterone levels. (A and B) Expression level of gastrocnemius *Ar* and *Vdr* by qPCR of SWT and SKO mice treated calcipotriol or vehicle (*n* = 6-8). (C and D) Representative western blotting results showing the analysis of AR and VDR in gastrocnemius of male (C) and female (D) mice (*n* = 6 biological replicates per experimental group). (E) The serum testosterone level of male and female mice (*n* = 6-8). (F) Western Blotting results showing AR and VDR in gastrocnemius of SWT male and female mice (*n* = 4 biological replicates per experimental group). Data are represented as mean ± SEM. *, †*p* < 0.05, ***p* < 0.01, and ***, †††*p* < 0.001 by two-way ANOVA (two-tailed) with Tukey’s *post hoc* for multiple comparisons or by Student’s unpaired two-tailed *t*-test. † Main effect of SWT vs. SKO. veh, vehicle; cal, calcipotriol.

**Figure S7**


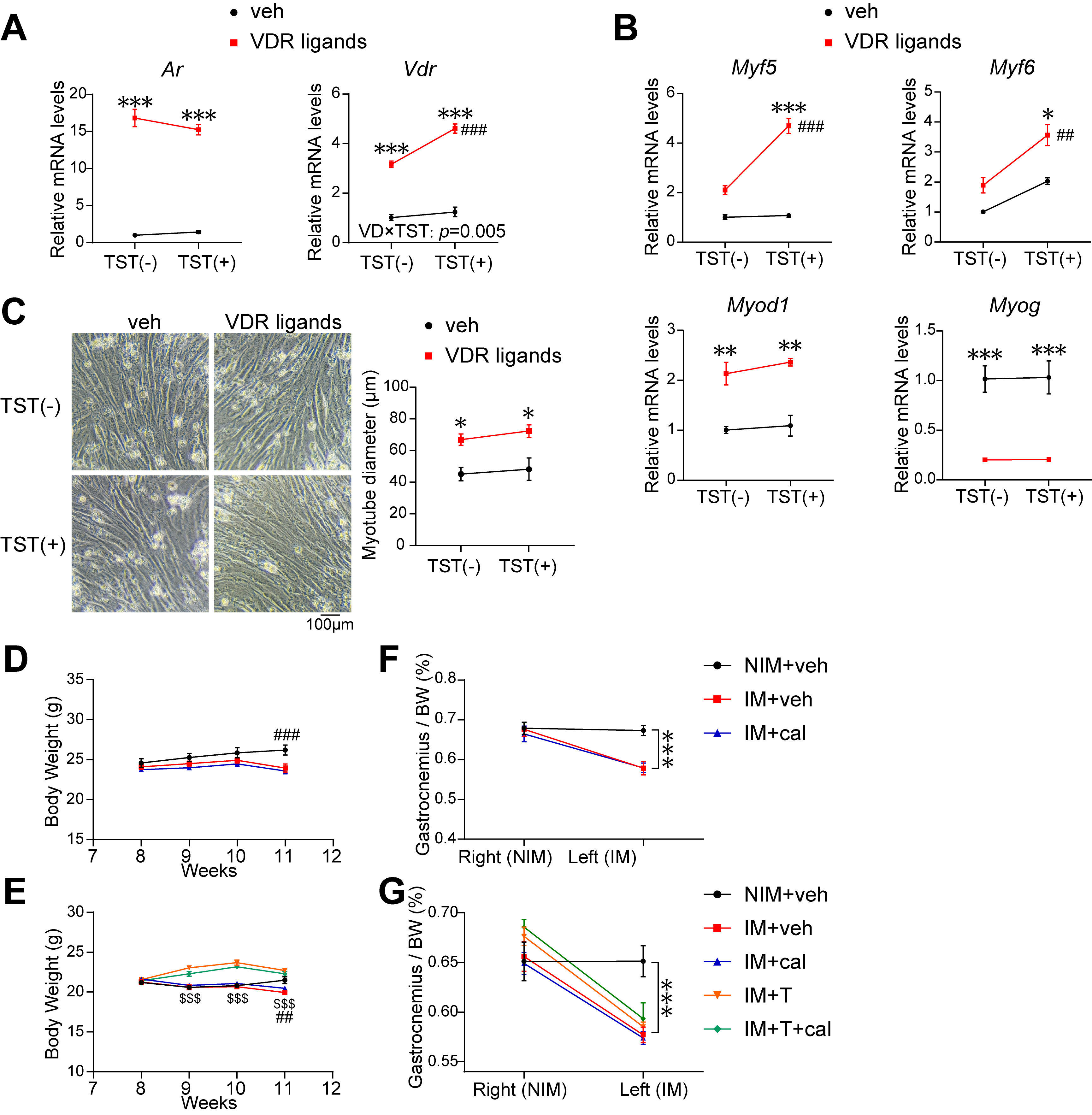


**Figure S7** Testosterone influences the effects of vitamin D on muscle. (A and B) Expression level of *Ar*, *Vdr* (A) and myofiber differentiation genes (B) measured by qPCR. (C) Representative images of C2C12 myotubes, and the diameter of myotubes was measured. Scale bars, 100 μm. Differentiation of C2C12 cells was induced by 2% horse serum plus every two days’ treatment of testosterone, VDR ligands, or vehicle for 8 days (*n* = 3). [* VDR ligands vs. vehicle, # TST (+) vs. TST (-)]. (D-G) Levels of body weight (B, C) and gastrocnemius muscle mass (D, E) in mice from the sham surgery and castration groups after intervention (n = 6-10). [$ IM+veh vs. IM+T, # IM+veh vs. NIM+veh]. Data are represented as mean ± SEM. **p* < 0.05, ##*p* < 0.01, and $$$, ###, ****p* < 0.001 by two-way ANOVA (two-tailed) with Tukey’s *post hoc* for multiple comparisons. veh, vehicle; cal, calcipotriol; IM, immobilization; NIM, non-immobilization; TST, testosterone; T or TSTE, testosterone enanthate.

**Table S1** Descriptive characteristics of the study population stratified by serum 25(OH)D_3_ level

| Characteristic | Serum 25(OH)D_3_, nmol/L | | | *p* for  trend |
| --- | --- | --- | --- | --- |
|  | Total | < 50 | ≥ 50 |  |
| N | 5707 | 2784 | 2923 |  |
| Sex |  |  |  | <0.001 |
| Male, n (%) | 3095 (54.2) | 1419 (51.0) | 1676 (57.3) |  |
| Female, n (%) | 2612 (45.8) | 1365 (49.0) | 1247 (42.7) |  |
| Age, years | 42.0 [28.0, 58.0] | 41.0 [27.0, 56.0] | 43.0 [29.0, 60.0] | <0.001 |
| Body mass index, kg/m^2^ | 168.2 [160.8, 175.3] | 167.3 [160.2, 174.3] | 169.1 [161.6, 176.2] | <0.001 |
| Race |  |  |  | <0.001 |
| Mexican American, n (%) | 810 (14.2) | 429 (15.4) | 381 (13.0) |  |
| Other Hispanic, n (%) | 620 (10.9) | 271 (9.7) | 349 (11.9) |  |
| Non-Hispanic White, n (%) | 2091 (36.6) | 583 (20.9) | 1508 (51.6) |  |
| Non-Hispanic Black, n (%) | 1421 (24.9) | 1052 (37.8) | 369 (12.6) |  |
| Non-Hispanic Asian, n (%) | 578 (10.1) | 363 (13.0) | 215 (7.4) |  |
| Other Race, n (%) | 187 (3.3) | 86 (3.1) | 101 (3.5) |  |
| Smoking status |  |  |  | 1.000 |
| Yes, n (%) | 1621 (28.4) | 791 (28.4) | 830 (28.4) |  |
| No, n (%) | 4086 (71.6) | 1993 (71.6) | 2093 (71.6) |  |
| Education level |  |  |  | 0.021 |
| Less than 9th grade, n (%) | 454 (8.0) | 220 (7.9) | 234 (8.0) |  |
| 9-11th grade, n (%) | 982 (17.2) | 498 (17.9) | 484 (16.6) |  |
| High school graduate/GED or equivalent, n (%) | 1399 (24.5) | 686 (24.6) | 713 (24.4) |  |
| Some college or AA degree, n (%) | 1784 (31.3) | 897 (32.2) | 887 (30.3) |  |
| College graduate or above, n (%) | 1088 (19.1) | 483 (17.3) | 605 (20.7) |  |
| Physical activity |  |  |  | <0.001 |
| Inactive, n (%) | 2502 (43.8) | 1330 (47.8) | 1172 (40.1) |  |
| Moderate, n (%) | 1210 (21.2) | 630 (22.6) | 580 (19.8) |  |
| Active, n (%) | 1995 (35.0) | 824 (29.6) | 1171 (40.1) |  |
| Serum calcium, mmol/L | 2.35 [2.30, 2.40] | 2.35 [2.30, 2.40] | 2.35 [2.30, 2.42] | <0.001 |
| Serum 25(OH)D_3_, nmol/L | 52.9 (22.9) | 34.5 (9.9) | 70.4 (17.3) | <0.001 |
| Serum testosterone, ng/dL | 203.0 [22.5, 414.5] | 138.0 [21.6, 397.3] | 236.0 [23.6, 426.0] | <0.001 |
| Combined grip strength, kg | 71.0 [56.1, 89.7] | 69.3 [55.2, 88.0] | 72.6 [57.3, 91.4] | <0.001 |

Continuous variables are expressed as the mean ± standard deviation (SD) or median and interquartile range (IQR), and categorical variables are expressed as frequencies (%). Continuous variables were analyzed Student’s unpaired two-tailed t-test, and categorical variables were analyzed by the *χ^2^* test.

**Table S2** Descriptive characteristics of the study population stratified by sex

|  | Total | Male | Female | *p* for trend |
| --- | --- | --- | --- | --- |
| N | 5707 | 3095 | 2612 |  |
| Age, years | 42.0 [28.0, 58.0] | 42.0 [29.0, 59.0] | 42.0 [27.0, 57.0] | 0.128 |
| Body mass index, kg/m^2^ | 168.2 [160.8, 175.3] | 174.0 [169.1, 179.7] | 160.9 [156.1, 165.6] | <0.001 |
| Race |  |  |  | 0.003 |
| Mexican American, n (%) | 810 (14.2) | 441 (14.2) | 369 (14.1) |  |
| Other Hispanic, n (%) | 620 (10.9) | 314 (10.1) | 306 (11.7) |  |
| Non-Hispanic White, n (%) | 2091 (36.6) | 1183 (38.2) | 908 (34.8) |  |
| Non-Hispanic Black, n (%) | 1421 (24.9) | 722 (23.3) | 699 (26.8) |  |
| Non-Hispanic Asian, n (%) | 578 (10.1) | 322 (10.4) | 256 (9.8) |  |
| Other Race, n (%) | 187 (3.3) | 113 (3.7) | 74 (2.8) |  |
| Smoking status |  |  |  | <0.001 |
| Yes, n (%) | 1621 (28.4) | 1044 (33.7) | 577 (22.1) |  |
| No, n (%) | 4086 (71.6) | 2051 (66.3) | 2035 (77.9) |  |
| Education level |  |  |  | <0.001 |
| Less than 9th grade, n (%) | 454 (8.0) | 260 (8.4) | 194 (7.4) |  |
| 9-11th grade, n (%) | 982 (17.2) | 541 (17.5) | 441 (16.9) |  |
| High school graduate/GED or equivalent, n (%) | 1399 (24.5) | 824 (26.6) | 575 (22.0) |  |
| Some college or AA degree, n (%) | 1784 (31.3) | 868 (28.0) | 916 (35.1) |  |
| College graduate or above, n (%) | 1088 (19.1) | 602 (19.5) | 486 (18.6) |  |
| Physical activity |  |  |  | <0.001 |
| Inactive, n (%) | 2502 (43.8) | 1149 (37.1) | 1353 (51.8) |  |
| Moderate, n (%) | 1210 (21.2) | 624 (20.2) | 586 (22.4) |  |
| Active, n (%) | 1995 (35.0) | 1322 (42.7) | 673 (25.8) |  |
| Serum calcium, mmol/L | 2.35 [2.30, 2.40] | 2.35 [2.30, 2.42] | 2.35 [2.28, 2.40] | <0.001 |
| Serum 25(OH)D_3_, nmol/L | 52.9 (22.9) | 53.7 (21.4) | 51.9 (24.5) | <0.001 |
| Serum testosterone, ng/dL | 203.0 [22.5, 414.5] | 395.0 [289.9, 523.0] | 21.3 [14.1, 30.9] | <0.001 |
| Combined grip strength, kg | 71.0 [56.1, 89.7] | 88.1 [76.1, 99.9] | 56.6 [49.2, 64.2] | <0.001 |

Continuous variables are expressed as the mean ± standard deviation (SD) or median and interquartile range (IQR), and categorical variables are expressed as frequencies (%). Continuous variables were analyzed Student’s unpaired two-tailed t-test, and categorical variables were analyzed by the *χ^2^* test.

**Table S3** Linear regression analysis of grip strength in adults

| Characteristic | Grip strength | |
| --- | --- | --- |
|  | B (SEM) | *p* value |
| **Model 1** |  |  |
| Age | -0.23 (0.01) | <0.001 |
| Body mass index | 0.99 (0.02) | <0.001 |
| VD | -1.18 (0.60) | 0.051 |
| TST | 3.78 (0.65) | <0.001 |
| VD×TST | 0.33 (0.13) | 0.010 |
| **Model 2** |  |  |
| Age | -0.20 (0.01) | <0.001 |
| Body mass index | 0.99 (0.02) | <0.001 |
| Race |  |  |
| Other Hispanic | -1.79 (0.72) | 0.013 |
| Non-Hispanic White | -3.11 (0.61) | <0.001 |
| Non-Hispanic Black | 0.03 (0.63) | 0.967 |
| Non-Hispanic Asian | -3.75 (0.77) | <0.001 |
| Other Race | -2.36 (1.11) | 0.033 |
| Education level |  |  |
| 9-11th grade | 0.06 (0.79) | 0.935 |
| High school graduate/GED or equivalent | 1.16 (0.76) | 0.129 |
| Some college or AA degree | 0.69 (0.76) | 0.360 |
| College graduate or above | 1.35 (0.82) | 0.097 |
| Physical activity | 1.88 (0.21) | <0.001 |
| Smoking status | -0.95 (0.42) | 0.022 |
| Serum calcium | 0.08 (2.07) | 0.997 |
| VD | -0.87 (0.60) | 0.151 |
| TST | 3.62 (0.64) | <0.001 |
| VD×TST | 0.32 (0.13) | 0.011 |

Model 1 was adjusted for age and BMI; Model 2 was adjusted for the variables included in Model 1 + race, education level, physical activity, smoking status, and serum calcium level. Testosterone and 25(OH)D_3_ were processed using log-transformed and box-cox-transformed variables, respectively. Abbreviations: VD: Serum 25(OH)D_3_; TST, Serum testosterone.

**Table S4** Linear regression analysis of grip strength stratified by testosterone in adults

|  | TST < 300 ng/dL | |  | TST ≥ 300 ng/dL | |
| --- | --- | --- | --- | --- | --- |
| Characteristic | B (SEM) | *p* value |  | B (SEM) | *p* value |
| **Model 1** |  |  |  |  |  |
| Age | -0.17 (0.01) | <0.001 |  | -0.33 (0.02) | <0.001 |
| Body mass index | 1.39 (0.02) | <0.001 |  | 0.94 (0.04) | <0.001 |
| Serum 25(OH)D_3_ | -0.10 (0.25) | 0.681 |  | 1.21 (0.38) | 0.001 |
| **Model 2** |  |  |  |  |  |
| Age | -0.15 (0.01) | <0.001 |  | -0.31 (0.02) | <0.001 |
| Body mass index | 1.39 (0.03) | <0.001 |  | 0.93 (0.04) | <0.001 |
| Race |  |  |  |  |  |
| Other Hispanic | -1.15 (0.90) | 0.202 |  | -2.36 (1.30) | 0.070 |
| Non-Hispanic White | -4.63 (0.78) | <0.001 |  | -2.87 (1.08) | 0.008 |
| Non-Hispanic Black | -1.79 (0.78) | 0.022 |  | 1.27 (1.14) | 0.265 |
| Non-Hispanic Asian | -1.58 (0.78) | 0.104 |  | -5.61 (1.34) | <0.001 |
| Other Race | -2.38 (1.45) | 0.102 |  | -3.95 (1.86) | 0.034 |
| Education level |  |  |  |  |  |
| 9-11th grade | -1.25 (0.99) | 0.210 |  | 0.99 (1.41) | 0.482 |
| High school graduate/GED or equivalent | -0.29 (0.96) | 0.764 |  | 2.53 (1.35) | 0.062 |
| Some college or AA degree | -1.72 (0.94) | 0.067 |  | 2.45 (1.37) | 0.074 |
| College graduate or above | -1.31 (1.02) | 0.200 |  | 2.54 (1.45) | 0.082 |
| Physical activity | 2.26 (0.28) | <0.001 |  | 1.98 (0.37) | <0.001 |
| Smoking status | -0.64 (0.55) | 0.239 |  | -1.56 (0.67) | 0.021 |
| Serum calcium | 3.19 (2.59) | 0.219 |  | -4.37 (3.85) | 0.257 |
| Serum 25(OH)D_3_ | -0.40 (0.25) | 0.116 |  | 0.78 (0.39) | 0.042 |

Model 1 was adjusted for age and BMI; Model 2 was adjusted for the variables included in Model 1 + race, education level, physical activity, smoking status, and serum calcium level.

**Table S5** qPCR primer sequences.

| Genes* | Forward, 5’-3’ | Reverse, 5’-3’ |
| --- | --- | --- |
| hVDR | TACTGCTGAAGTCAAGTGCCAT | TCCGGCTTTGGTCACGTC |
| hCYP2R1 | CCATTCCTAAAGGCACAACAGT | AATCGCTCAGGATGGAACAC |
| hCYP3A4 | AAGGGATGGCACCGTAAGTG | GGTCTCTGGTGTTCTCAGGC |
| hGAPDH | CTCAAGATCATCAGCAATGCCT | GGTCATGAGTCCTTCCACGAT |
| mVdr | TGGCTTCAGGGACCTCACCTC | ACATGATCACCTCAATGGCACT |
| mCyp2r1 | CGGGTGTATGGCGAGATTTT | AAGGAAGGCATGGTCTATCTG |
| mCyp3a41 | CAGTGGAAAACTCAAGGAGATGT | CTTGCCTTTCTCTGCCTCT |
| mCyp27b1 | TTGTCCCAGATCTTGACCCA | GGGTCATGGGCTTGATAGGA |
| mCyp24a1 | ACGGTAGGCTGCTGAGATTT | TCTGGTCCCTGAAGTTCACC |
| mGapdh | TTGGGCTACACTGAGGACCA | GCCGTATTCATTGTCATACCAGG |
| mMyh1 | TACTCACGCCAGCTAGACGAA | GCACTCTTGGCCTTTATCTCC |
| mMyh2 | AGCGACTGATCAACGACCTG | TGCCTGGAAAATTCACCGGAT |
| mMyh4 | AACAGAAGCGCAACATCGAA | CTCCTCGGTCTGGTAGGTGA |
| mMyh7 | TCAGCCATGCCAACCGTA | ATGTTCTCTTTCAGGTCGTCA |
| mIl-1b | AGGTCAAAGGTTTGGAAGCA | TGAAGCAGCTATGGCAACTG |
| mIl-6 | TGGTACTCCAGAAGACCAGAGG | AACGATGATGCACTTGCAGA |
| mTnf | ACTCAAATGGGCTTTCCGAA | GACAGAGGCAACCTGACCAC |
| mIl20rb | AACCGAAATGCAACTGTCCT | AGGTCTTCCAGCTCAATAACCA |
| mCasp3 | ACAGCACCTGGTTACTATTCCT | CTCGAATTCCGTTGCCACC |
| mSerpina3n | CTCTCAGGTGGTCCACAAGG | ATCATTATCAGGAAAGGCCGAT |
| mMyod1 | TCCTCATAGCACAGGGGTGA | GCAAGCTGTGGGGAAAAGTG |
| mMyog | CCAACCCAGGAGATCATTTGC | ATATCCTCCACCGTGATGCT |
| mMyf5 | GCATGCCTGAATGTAACAGCC | CGTGATCCGATCCACAATGCT |
| mMyf6 | CCCACAGATCGTCGGAAAGCA | CCACAGTCCGACGCTTCAGG |
| mPax7 | GCGTCCAGGTCTGGTTCAGT | AAACCTCCCGGCAGAAGGTG |
| mFbxo32 | ACATCATGCAGAGGCTGAGT | GCTTGCGAATCTGCCTCTCTGA |
| mTrim63 | CAAGAGCATTGTAGAAGCCTCC | TCCCAAAGTCAATGGCCCTC |
| mPpargc1a | AATTGAAGAGCGCCGTGTGA | AGCGGTCTCTCAATTCTGTCC |
| mTomm20 | GCTTCCGACCATTAGTCAGAG | TATCTTGTTGGTATCTGGCTCA |
| mUcp3 | TGTTTACTGACAACTTCCCCTG | AGCGTTCATGTATCGGGTCT |
| mAr | CACAAGTCTCGGATGTACAGC | GCCCATCCACTGGAATAATGC |
| mSod1 WT | TGAACCAGTTGTGTTGTCAGG | TCCATCACTGGTCACTAGCC |
| mSod1 KO | CTTCTCGTCTTGCTCTCTCTGGT | CCTCGCACACATTCCACATCC |

* The lower letters of h and m were added as initials of the gene symbols to indicate the species of human and mouse, respectively.

**Table S6** List of antibodies used in this study.

| Name | Supplier | Identifier |
| --- | --- | --- |
| mouse anti-Beta Actin | proteintech | Clone: 7D2C10; Cat# 60008-1-Ig;  RRID: AB_2289225 |
| mouse anti-GAPDH | ABclonal | Clone: AMC0062R; Cat# AC002;  RRID: AB_2736879 |
| rabbit anti-Cytochrome P450 3A4 | abcam | Clone: EPR6202; Cat# ab124921;  RRID: AB_2924823 |
| rabbit anti-CYP3A4 | ABclonal | Cat# A2544; RRID: AB_2764433 |
| rabbit anti-Vitamin D Receptor | abcam | Clone: EPR4552; Cat# ab109234;  RRID: AB_10862713 |
| mouse anti- Myosin Heavy Chain Type IIB | DSHB | Cat# BF-F3; RRID: AB_2266724 |
| rabbit anti-SOD1 | Santa Cruz | Clone: FL-154; Cat# sc-11407;  RRID: AB_2193779 |
| rabbit anti-phospho-AKT (Ser473) | proteintech | Cat# 28731-1-AP; RRID: AB_2881201 |
| mouse anti- AKT | proteintech | Clone: 2C5D1; Cat# 60203-2-Ig;  RRID: AB_10912803 |
| rabbit anti-phospho-S6 Ribosomal protein (Ser235/236) | proteintech | Cat# 29223-1-AP; RRID: AB_2918253 |
| rabbit anti-S6 Ribosomal protein | proteintech | Clone: 6L9; Cat# 80208-1-RR;  RRID: AB_2918876 |
| rabbit anti-phospho-FoxO3a (Ser253) | bioss | Cat# bs-3140R; RRID: AB_10857054 |
| rabbit anti-FOXO3A | proteintech | Cat# 10849-1-AP; RRID: AB_2247214 |
| mouse anti- PGC-1α | Santa Cruz | Clone: 4A8; Cat# sc-517380;  RRID: AB_2755043 |
| rabbit anti-Beclin-1 | Cell Signaling | Clone: D40C5; Cat# 3495;  RRID: AB_10889247 |
| rabbit anti-LC3B | Cell Signaling | Cat# 2775; RRID: AB_915950 |
| rabbit anti-androgen receptor | proteintech | Cat# 22089-1-AP; RRID: AB_11182176 |
| goat anti-rabbit IgG, HRP-linked antibody | Cell Signaling | Cat# 7074; RRID: AB_2099233 |
| goat anti- mouse IgG, HRP-linked antibody | proteintech | Cat# SA00001-1; RRID: AB_2722565 |
| goat anti-mouse IgG H&L (Alexa Fluor® 488) | abcam | Cat# ab150113; RRID: AB_2576208 |

## Supplemental References

S1. Ethgen O, Beaudart C, Buckinx F, Bruyère O, Reginster JY. The Future Prevalence of Sarcopenia in Europe: A Claim for Public Health Action. *Calcified tissue international*. 2017;**100**:229-34.

S2. Wierzbicka A, Oczkowicz M. Sex differences in vitamin D metabolism, serum levels and action. *Br J Nutr*. 2022;**128**:2115-30.

S3. Fox FAU, Koch L, Breteler MMB, Ahmad Aziz N. 25-hydroxyvitamin D level is associated with greater grip strength across adult life span: a population-based cohort study. *Endocrine Connections*. 2023;**12**:e220501.

S4. Hashim PH, Kinnear HM, Cruz CD, Padmanabhan V, Moravek MB, Shikanov A. Pharmacokinetic comparison of three delivery systems for subcutaneous testosterone administration in female mice. *Gen Comp Endocrinol*. 2022;**327**:114090.

S5. Beer TM. ASCENT: the androgen-independent prostate cancer study of calcitriol enhancing taxotere. *BJU Int*. 2005;**96**:508-13.

S6. Nguyen HM, Corey E. Methodology to Investigate Androgen-Sensitive and Castration-Resistant Human Prostate Cancer Xenografts in Preclinical Setting. In: Saatcioglu F editor. Totowa, NJ: Humana Press; 2011. pp. 295-312.

S7. Xu H, Ranjit R, Richardson A, Van Remmen H. Muscle mitochondrial catalase expression prevents neuromuscular junction disruption, atrophy, and weakness in a mouse model of accelerated sarcopenia. *J Cachexia Sarcopenia Muscle*. 2021;**12**:1582-96.

S8. Arany Z, He H, Lin J, Hoyer K, Handschin C, Toka O, et al. Transcriptional coactivator PGC-1 alpha controls the energy state and contractile function of cardiac muscle. *Cell Metab*. 2005;**1**:259-71.

S9. Cruz-Jentoft AJ, Bahat G, Bauer J, Boirie Y, Bruyère O, Cederholm T, et al. Sarcopenia: revised European consensus on definition and diagnosis. *Age Ageing*. 2019;**48**:16-31.

S10. Chen SE, Jin B, Li YP. TNF-alpha regulates myogenesis and muscle regeneration by activating p38 MAPK. *Am J Physiol Cell Physiol*. 2007;**292**:C1660-71.

S11. Kim G, Lee SE, Lee YB, Jun JE, Ahn J, Bae JC, et al. Relationship Between Relative Skeletal Muscle Mass and Nonalcoholic Fatty Liver Disease: A 7-Year Longitudinal Study. *Hepatology (Baltimore, Md)*. 2018;**68**:1755-68.
